# Past climate change on Sky Islands drives novelty in a core developmental gene network and its phenotype

**DOI:** 10.1186/s12862-015-0448-4

**Published:** 2015-09-04

**Authors:** Marie-Julie Favé, Robert A. Johnson, Stefan Cover, Stephan Handschuh, Brian D. Metscher, Gerd B. Müller, Shyamalika Gopalan, Ehab Abouheif

**Affiliations:** Department of Biology, McGill University, 1205 Dr. Penfield avenue, Montréal, Québec, Canada; School of Life Sciences, Arizona State University, Tempe, AZ 85287-4501 USA; Museum of Comparative Zoology, Harvard University, 26 Oxford Street, Cambridge, MA 02138 USA; Department of Theoretical Biology, University of Vienna, Althanstrasse 14, Vienna, 1090 Austria

## Abstract

**Background:**

A fundamental and enduring problem in evolutionary biology is to understand how populations differentiate in the wild, yet little is known about what role organismal development plays in this process. Organismal development integrates environmental inputs with the action of gene regulatory networks to generate the phenotype. Core developmental gene networks have been highly conserved for millions of years across all animals, and therefore, organismal development may bias variation available for selection to work on. Biased variation may facilitate repeatable phenotypic responses when exposed to similar environmental inputs and ecological changes. To gain a more complete understanding of population differentiation in the wild, we integrated evolutionary developmental biology with population genetics, morphology, paleoecology and ecology. This integration was made possible by studying how populations of the ant species *Monomorium emersoni* respond to climatic and ecological changes across five ‘Sky Islands’ in Arizona, which are mountain ranges separated by vast ‘seas’ of desert. Sky Islands represent a replicated natural experiment allowing us to determine how repeatable is the response of *M. emersoni* populations to climate and ecological changes at the phenotypic, developmental, and gene network levels.

**Results:**

We show that a core developmental gene network and its phenotype has kept pace with ecological and climate change on each Sky Island over the last ∼90,000 years before present (BP). This response has produced two types of evolutionary change within an ant species: one type is unpredictable and contingent on the pattern of isolation of Sky lsland populations by climate warming, resulting in slight changes in gene expression, organ growth, and morphology. The other type is predictable and deterministic, resulting in the repeated evolution of a novel wingless queen phenotype and its underlying gene network in response to habitat changes induced by climate warming.

**Conclusion:**

Our findings reveal dynamics of developmental gene network evolution in wild populations. This holds important implications: (1) for understanding how phenotypic novelty is generated in the wild; (2) for providing a possible bridge between micro- and macroevolution; and (3) for understanding how development mediates the response of organisms to past, and potentially, future climate change.

**Electronic supplementary material:**

The online version of this article (doi:10.1186/s12862-015-0448-4) contains supplementary material, which is available to authorized users.

## Background

How populations differentiate within species is a fundamental problem in evolutionary biology that is key for uncovering the processes that generate biological diversity, including microevolution, speciation, and the emergence of phenotypic novelty [1–6]. Traditionally, biologists have used the tools of population and quantitative genetics, two fields central to the modern evolutionary synthesis, to study population differentiation [1, 7–9]. These fields have significantly advanced our knowledge of variation and change in the frequency of alleles and phenotypes (quantitative and discrete) within and between populations [10]. They have also uncovered signatures of natural selection and genetic drift and are being used to identify loci responsible for adaptive and non-adaptive phenotypes driving the evolution of populations [8, 11–14]. However, a largely unexplored dimension of this problem is what role, if any, does organismal development play in the process of population differentiation in the wild [3, 15, 16].

Organismal development integrates the action of gene regulatory networks with environmental inputs to generate the phenotype [3, 17–19], and therefore, may play a key role in facilitating phenotypic differentiation of populations and species exposed to ecological changes [3, 17, 19]. Major advances in the field of evolutionary developmental biology, such as the discovery of Hox genes, have revealed an unexpected degree of evolutionary conservation of developmental regulatory genes across the animal kingdom [7, 20]. These regulatory genes, which are transcription factors and signalling molecules, are assembled in hierarchically organized networks [18, 20]. A primary function of developmental networks is body plan formation [18, 20]. For example, the expression, structure, function, and regulation of the developmental genes *Sonic hedgehog* and *patched* have been conserved for hundreds of millions of years across vertebrates and invertebrates [21]. Both *Sonic hedgehog* and its downstream target *patched* regulate anterior/posterior patterning in the developing limb of both fruit flies and chickens [21]. The high degree of conservation and structure of developmental networks suggests that these networks may bias the variation that selection can act upon. Such bias may facilitate repeatable phenotypic responses when populations are exposed to similar environmental inputs or encounter similar ecological changes [1, 3, 22, 23]. Here we use an integrative approach that combines multiple levels of organization (gene networks, development, and phenotype) as well as multiple fields (population genetics, paleoecology and ecology, morphology, and evolutionary developmental biology) to gain a more complete understanding of population differentiation in the wild.

**Fig. 1:**
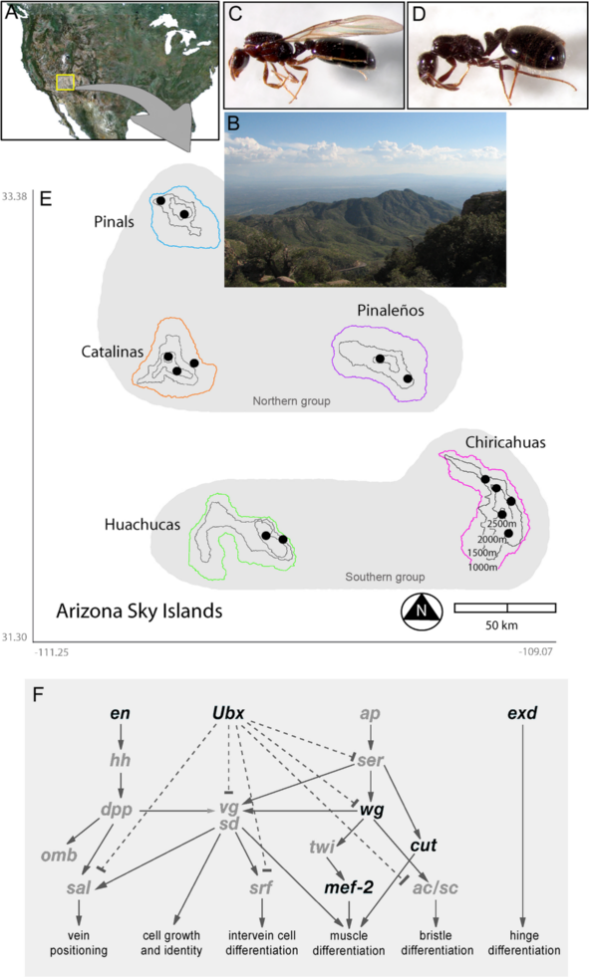
The Arizona Sky Islands. **a** Satellite image of North America showing the location of the Arizona Sky Islands (yellow box). **b** A picture taken on a Sky Island from high elevation. Across the desert at the horizon, a second Sky Island can be distinguished. Alternative phenotypes of *Monomorium emersoni* queens: **c** winged queen and **d** wingless queen. **e** Map of Southeastern Arizona showing the relative location of the five Sky Islands indicating our sampling sites (black dots) and 500m topographic contour lines. **f** Representation of the highly conserved gene network that controls wing development in *Drosophila melanogaster*. Arrows indicate activation and bars indicate repression. Genes examined in this study are shown in black

Our ability to integrate all of these levels and approaches in a single study was made possible by using recently diverged populations within the ant species *Monomorium emersoni* along five ‘Sky Islands’ in Arizona. Sky Islands are a group of mountain ranges in the American Southwest across the Arizona-Mexico border that are isolated by large areas of deserts with limited genetic exchange between them [24, 25]. From the perspective of the evolutionary biologist, Sky Islands represent a replicated natural experiment, in which each Sky Island is a natural laboratory of high evolutionary potential. Isolation of the Sky Islands has facilitated the evolution of several endemic species: several squirrels, plants, ants, more than 60 species of land snails, as well as many endangered species that are found only in the Sky Islands of Arizona [26–29]. In essence, this archipelego of Sky Islands is comparable to oceanic islands isolated by sea water, like those in the Galapagos, Hawaii and the Caribbean islands [27] (Fig. 1a and b).

The paleoecological record of the Sky Islands is one of the most complete records available [24, 25]. Numerous studies have reconstructed the major ecological and climatic changes that have occurred in the region by using the stratification of pollen grains and middens of pack rats in the fossil record. These studies show that during the Pleistocene glacial periods ∼90,000–20,000 years BP when atmospheric temperature around the region was substantially cooler, there was a largely continuous forest landscape connecting Sky Island mountain ranges [24, 25]. The region then began warming up between ∼20,000–10,000 years BP, and atmospheric temperatures increased substantially, resulting in the formation of vast deserts that isolated the Sky Islands [24, 25]. Species that occurred at lower elevations in the intervening valleys during the Pleistocene glacial periods, such as Ponderosa pine trees and Douglas fir, are currently only found at high-elevations. These past climatic changes in the region have generally caused shifts in elevation, expansions, and contractions of populations in numerous species [28–32]. Each Sky Island is now considered to be one of the steepest terrestrial ecological gradients in North America, from low-elevation deserts, through mid-elevation oak-juniper woodlands, to high elevation coniferous forests [27]. Several ecological factors vary steeply along these altitudinal clines, in particular temperature, precipitation and habitat fragmentation [33–35]. Environmental changes that would normally be observed across a latitudinal gradient stretching thousands of kilometres can be observed across a few hundred meters of elevational change in the Sky Islands [26, 27, 36]. This poses a unique adaptive challenge for organisms on Sky Islands that have distributional ranges that encompass more than one altitudinal zone [26, 27, 36]. These steep gradients therefore behave as powerful sensors of climatic and environmental changes because noticeable changes in communities occur rapidly and over short geographical distances [33–35].

Ants are an ecologically dominant group [37] that have long been used as bioindicators of environmental change [38, 39] and climate warming [40, 41]. In most ant species, the colony is made up of a single winged queen that performs most of the reproduction in the colony and her wingless workers that perform almost all the other tasks [37]. Throughout most of the year, the queen produces eggs that develop into wingless workers, but in response to specific environmental conditions associated with spring time, such as the gradual increase in daylight and temperature, they begin producing virigin queens and males that use their fully functional wings to disperse and participate in mating flights away from the mother colony. Immediately after these mating flights, males die while queens tear off their wings to found a new nest underground [37]. This life history strategy is known as ‘independent colony foundation’ [42–45]. In some ant species, however, an alternative life history strategy called ‘dependent colony foundation’ has evolved [42–45]. In these species, there are multiple queens in a single ant colony in which some or all of the queens are wingless. Virgin queens that are wingless mate on the ground and disperse on foot aided and accompanied by wingless workers from the mother colony. This strategy dramatically decreases mortality of wingless queens, but at the expense of long distance dispersal. Wingless queens have evolved multiple times independently within ants [43–45], suggesting that wingless queens represent an adaptive life-history strategy. It takes less energy to produce wingless queens, and selection may favor the evolution of wingless queens in particular ecological conditions where aerial dispersal is risky [29, 46, 47].

Colonies of the ant species *M. emersoni* are distributed throughout the Sky Islands, where we discovered the presence of two alternative queen phenotypes, winged and wingless (Fig. 1c and d) [48]. A single *M. emersoni* colony can be headed by one or multiple queens, ranging from 1 to more than 70, and colonies can be composed either exclusively by winged queens, exclusively by wingless queens, or a mixture of winged and wingless queens [48]. Current evidence suggests that determination of alternative queen phenotypes in a colony occurs during development and can be influenced by both environmental and genetic factors [43, 49, 50]. On each Sky Island, *M. emersoni* occurs sparsely at lower elevations, where its abundance is very low and is associated with creek or woody patches near mountain ranges [48]. At mid-elevations of each Sky Island, from ∼1500–2300 meters, *M. emersoni* occurs at high densities in oak-juniper woodlands, whereas at the highest elevations of each Sky Island, from 2300 m and above, *M. emersoni* occurs in patches of high density in coniferous forests dominated by Pinyon pine and Douglas fir [48].

Studying how population differentiation occurs in *M. emersoni* along several naturally replicated ecological gradients on five Sky Islands in Arizona provides a unique opportunity to ask whether organismal development facilitates repeatable, and thus, predictable responses to similar ecological and climate changes on each Sky Island. Furthermore, the interaction between ecology and organismal development in the context of Sky Islands is key for understanding population dynamics of gene networks and its impacts on phenotypic evolution on microevolutionary timescales, as well as for understanding how past changes in developmental gene networks may affect the future course of evolution in response to climate change.

## Results

**Fig. 2:**
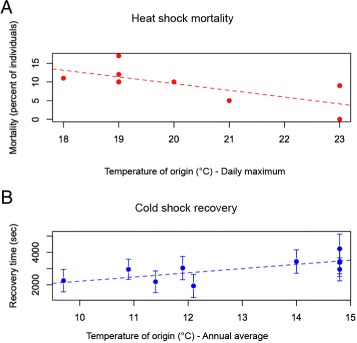
*M. emersoni* populations have adapted physiologically to the prevalent environmental conditions that were established after climate warming began 10,000 years BP. **a** Heat shock experiment: *M. emersoni* workers from warmer collection sites show significantly less mortality after a one hour heatshock at 50 ° C than workers from colder collection sites (R^2^ = 0.42, *p* = 0.035), indicating that *M. emersoni* colonies are adapted to the local environmental conditions that were established after climatic warming. **b** Cold shock experiment: *M. emersoni* workers from colder collection sites show a significantly more rapid recovery after a 16h cold shock at 4 ° C than workers from warmer collection sites (R^2^ = 0.46, *p* = 0.018)

**Fig. 3:**
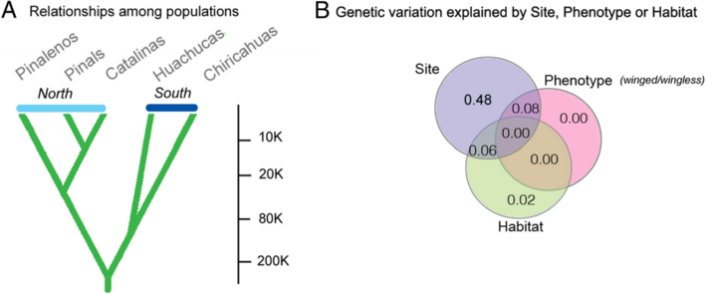
*M. emersoni* populations have been isolated from each other over the last 200,000 years and modern population structure reflects geography. **a** Simulations showing approximate timing of isolation events between *M. emersoni* populations in the Arizona Sky Islands during the last 200,000 years (see also Additional file 1: Figure S2). These simulations revealed two geographical groupings of Sky Islands: North and South. **b** Redundancy analysis showing that most of the mtDNA genetic variation is explained by geography (site). The Venn diagram represents the percentage of the genetic variation that can be attributed to each factor (circles) or their combined effect (overlapping areas). This analysis supports the hypothesis of multiple origins of the wingless phenotype, where genetic variation is structured by geography rather than habitat, phenotype or elevation (see also Additional file 1: Figure S3 and S4)

We first used heat and cold shock experiments to determine whether colonies of *M. emersoni* have responded to the establishment of warmer conditions at the lower limit of their elevational range across all 5 Sky Islands during the last 10,000 years BP. We found that colonies from warmer sites show increased tolerance to high temperature, whereas colonies from colder sites show increased tolerance to cold temperature (Fig. 2a and b). This suggests that individuals in colonies of *M. emersoni* have adapted to local temperature conditions along the ecological gradients. To determine how populations have changed their distribution over the last 100,000 years BP (Fig. 1e, Additional file 1: Table S3), we reconstructed historical levels of gene flow using mitochondrial (mtDNA, CO1 and CO2) and nuclear (*hymenotpaecin*) sequences (Additional file 1: Table S4 and Additional file 1: Figure S1). Our coalescent (Additional file 1: Figure S1) and evolutionary (Additional file 1: Figure S2) simulations show a sequential model of isolation between adjacent Sky Islands. A first split between southern (Huachucas and Chiricahuas) and northern (Pinaleños, Catalinas, and Pinals, Fig 1e and 3a) Sky Islands occurred around 80,000 years BP and was followed by the complete isolation of the 5 mountain ranges between 20,000 and 10,000 years BP (Additional file 1: Table S5, Figure S1 and S2). These results indicate that some Sky Island populations share a more recent history than others, but were subsequently isolated from one another during climatic warming. Together, these results show that *M. emersoni* populations have responded directly to climate warming by adapting physiologically and modifying their distribution.

We discovered two alternative queen phenotypes, winged and wingless, which likely reflect different life histories (independent and dependent colony foundation) adapted to different habitats. We therefore determined whether the frequency of winged and wingless phenotypes changes along the ecological gradients on each Sky Island. We found that wingless queens do indeed differ in their frequencies along the ecological gradient (Additional file 1: Figure S4 and S5), suggesting that there may be a set of environmental factors that the winged and wingless phenotypes may be adapting to on each Sky Island. To potentially explain the distribution of winged and wingless queens across collection sites, we used previous literature to construct 12 biological models (Table 1). Each model included one or several of 5 key environmental factors as well as possible interactions between those factors (Additional file 1: Table S1). We tested the ability of the 12 competing models to describe the data using a model-ranking approach [51]. Model m06, which includes the combined effects of habitat fragmentation and elevation, is by far the model with the highest probability (0.58) accounting for 50 % of the variability in the data (Table 1, Additional file 1: Table S2). Four out of the 5 highest ranking models included habitat fragmentation and/or elevation as explanatory variables, bringing additional support for a combined effect of these variables on the distribution of wingless queens along the ecological clines. Indeed, significant habitat fragmentation has been postulated to favor the evolution of wingless queens for short distance dispersal (dependent colony foundation) over winged queens for long distance dispersal (independent colony foundation), because winged queens have increased chances of landing in an inhospitable habitat. These results support a scenario where climate warming indirectly drove the evolution of the wingless queen phenotype through habitat changes. The paleoecological record indicates that during the last glaciation ∼90,000 to 20,000 years BP forests were ubiquitous and continuously distributed at the lower elevations [24, 25]. Because winged queens occur more frequently in low elevation and more continuous habitats, we can infer that the ancestral populations were likely composed of winged queens. This is consistent with our finding of significant gene flow between all five Sky Islands ∼90,000 years BP (Additional file 1: Figure S1). Subsequently, wingless queens emerged when climatic warming drove the appearance of fragmented high elevation habitats on each of the Sky Islands ∼20,000 years BP.

**Table 1:** A combination of landscape fragmentation and elevation best explain the distribution of wingless queens along ecological gradients. Based on previous studies, we generated 12 models (M01 to M12) representing alternative hypotheses to explain the variation in frequency of wingless queens along the ecological gradient of each Sky Islands. The 12 models were ranked using the method of [121] implemented in R (Additional file 1: Table S2) and model probabilities (Prob) are reported, with m06 scoring as the highest ranking model

Model	Formula	Prob	Underlying biological hypotheses
m01	Elev	0.03	Flightlessness in insects has been reported to increase with altitude [47, 127].
			Elevation influences a wide range of ecological factors and may account for their joint effects.
			Atmospheric pressure is lower at higher altitude and efficient flight may require costly adaptations [128].
m02	Temp	0.02	Insects will experience a decrease in annual thermal budget for growth and development. Critical temperature thresholds for growth, development and activity will be exceeded less frequently [128].
m03	Seas	0.02	Temperature seasonality, the variability in temperature over the year, is indicative a less stable environment. Flightless insects are known to be found in higher frequencies in more stable habitats [47].
m04	Frag	0.11	Flying insects will have a tendency to be blown away from patchy habitats, thus only wingless forms remain [46, 47]. The probability of finding a suitable habitat is decreased when habitats are patchy, thus dispersal risk is increased [129].
m05	Prod	0.17	The need for dispersal in high productivity habitats is reduced because the conditions for survival are met and the relative risk of long distance dispersal becomes too high [130]
m06	Frag + Elev	0.56	Both fragmentation and elevation influence the distribution of wingless queens.
m07	Frag + Elev + Frag x Elev	0.03	The effect of fragmentation depends on elevation: fragmentation may only be affecting the distribution of wingless queens at certain altitudes.
m08	Frag + Temp	0.03	Both fragmentation and temperature may influence the distribution of wingless queens.
m09	Frag + Temp + Frag x Temp	0.00	The effect of fragmentation depends on temperature: fragmentation may only be affecting the distribution of wingless queens at certaine temperature. Habitat fragmentation and temperature are know to be potentially interrelated [131].
m10	Frag + Prod	0.02	Both fragmentation and productivity can influence the distribution of wingless queens.
m11	Frag + Prod + Frag x Prod	0.00	Habitat fragmentation can affect productivity. Fragmentation may affect wingless queen distribution in habitats with a certain productivity level only. Habitat fragmentation and biomass growth are know to be potentially interrelated [131].
m12	Elev + Temp + Prod + Frag + Seas	0.00	Structure-rich model, including the effects of elevation, temperature, productivity, fragmentation and seasonality. Interactions were omitted.

Parameters abbreviations are Elev: elevation, Temp: temperature, Seas: seasonality, Frag: fragmentation, Prod: productivity. An (x) in the formula indicate the interaction between two variables

Our data show that emergence of the wingless queen phenotype occurred repeatedly by parallel evolution in response to similar ecological conditions arising on each of the Sky Islands during climatic warming. First, modern gene flow between mountain ranges is absent or low; the degree of genetic differentiation (*F*_*ST*_) is high between collection sites on different Sky Islands, but is lower within a Sky Island (Additional file 1: Table S3 and S5). Second, phylogenetic and redundancy analyses based respectively on multilocus AFLP data and mtDNA sequences show that *M. emersoni* colonized each Sky Island independently (Fig. 3b, Additional file 1: Figure S3 and S4). We find that populations on each Sky Island contain unique haplotype variants, while no shared haplotypes were recovered among Sky Islands, indicating that unique haplotypes originated within each Sky Island (Additional file 1: Figure S3). Finally, our results show that geography dictates the patterns of genetic differentiation at neutral loci: winged and wingless queens from the same Sky Island share a more recent ancestor than queens from different Sky Islands (Additional file 1: Figure S3 and S4), and genetic variation among queens is best explained by geography (collection site) rather than habitat or phenotype (Fig. 3b). All together, this supports an incipient process of parallel differentiation of the populations and suggests that *M. emersoni* wingless queens emerged independently on each Sky Island when each population encountered similar but newly established ecological conditions, namely, fragmented and high elevation habitats.

In many taxa, the repeated evolution of a trait observed across species within a genus, such as winglessness in stick insects [52], *Drosophila* wing spots [53], or armor plate loss in freshwater sticklebacks [1], have not only been generated by similar selective pressures but also by ancestral developmental potentials [3, 54–56]. Developmental potentials are unexpressed genetic programs, such as gene networks, or unexpressed developmental thresholds that determine sensitivity of these networks to specific environmental conditions. Developmental potentials are ancestral because they are often retained for millions of years in modern groups of organisms, most probably because of pleiotropy [3, 57]. In ants, Rajakumar et al. [54] demonstrated that ancestral developmental potentials can be induced by mutations or environmental perturbations to produce ancestral phenotypic variants (anomalies) that selection acts on to facilitate parallel evolution. We therefore searched the literature for evidence of an ancestral developmental potential to produce wingless queens in the genus *Monomorium*. We found that, unlike other genera, a large proportion of New World species (10 out of 16) in *Monomorium* show variable frequencies of appearance of wingless queens (Table 2). In 5 species (*M. emersoni*, *M. ergatogyna*, *M. trageri*, *M. viridae* and in *M. sp. AZ-03*), queens are winged but the existence of wingless phenotypes have been reported either as anomalies (sporadic occurrences) or alternative phenotypes (common occurrence of both phenotypes), while in the other five species (*M. compressum*, *M. cyaneum*, *M. ebeninum*, *M. sp. AZ-01* and *M. sp. cf. ergatogyna*), wingless queens appear as a fixed phenotype in the colony. The 6 remaining species are known to have winged queens only. The common occurrence of wingless queens as sporadic anomalies in colonies, as well as the evolution of species with alternative or fixed phenotypes, indicates the existence of an ancestral developmental potential to produce wingless queens in *Monomorium*, which likely played a role in facilitating the parallel evolution of the wingless queen phenotype within *M. emersoni*. This developmental potential to produce wingless queens could be unique to the genus *Monomorium*, or may be a more ancient feature that facilitated the ∼200 independent evolutionary origins of wingless queens across ants. The ancestral developmental potential to produce wingless queens may have been co-opted from the wing polyphenism in ants - the ability of a single genome to produce winged queens and wingless workers in a colony in response to environmental cues [58], just after the origin of ants. It is unlikely, however, that wingless queens evolved by directly co-opting this capacity to produce the wingless worker caste *de novo* during the evolution of *M. emersoni*, because wingless workers in *M. emersoni* interrupt wing development much earlier in development than wingless queens (Additional file 1: Figure S5). Collectively, our results suggest that in response to climatic change, selection on the phenotypic expression of the ancestral potential for producing wingless queens may have driven the parallel evolution of the wingless phenotype on each Sky Island.

**Table 2:** The presence of wing loss in queens in *Monomorium minimum*-group species. Summary of known queen phenotypes for *Monomorium minimum*-group species that are native to the United States and Mexico; species are listed alphabetically

Species	Queen phenotypes
*M. compressum*	Wingless ^a^
*M. cyaneum*	Wingless ^a^
*M. ebeninum*	Wingless ^a^
*M. emarginum*	Winged ^a^
*M. emersoni*	Winged and wingless ^c,d^
*M. ergatogyna*	Winged and wingless ^a,b^
*M. inquilinum*	Winged ^a^
*M. marjoriae*	Winged ^a^
*M. minimum*	Winged ^a^
*M. pergandei*	Winged ^a^
*M. talbotae*	Winged ^a^
*M. trageri*	Winged and wingless ^a^
*M. viride*	Winged and wingless ^a,c^
*M. sp. AZ-01*	Wingless ^c,d^
*M. sp. AZ-03*	Winged and wingless ^d^
*M. sp. cf. ergatogyna*- Baja	Wingless ^b,s^

^a^DuBois (1986)

^b^P.S. Ward, pers. obs.

^c^S.P. Cover, pers. obs.

^d^R.A. Johnson, pers. obs.

**Fig. 4:**
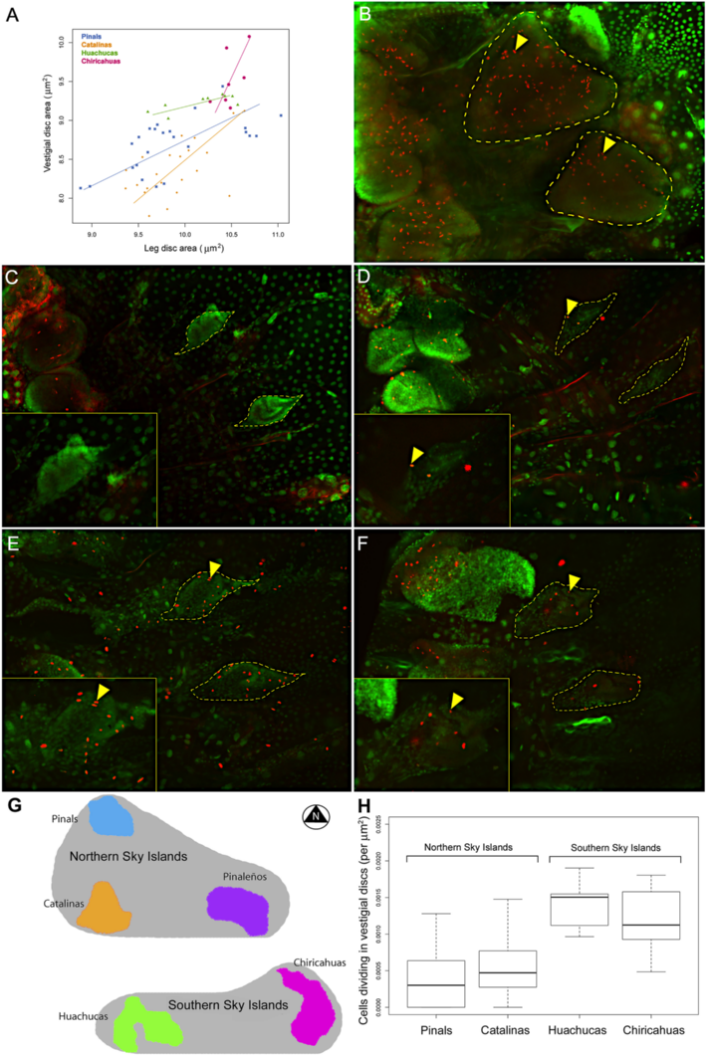
Wingless queens from the Northern and the Southern Sky Islands show different growth patterns. **a** Surface area (*μ*m^2^) of leg discs (x-axis) versus vestigial wing disc (y-axis) for wingless queen last instar larvae from different mountain ranges. A line was fitted through with a Standardized Major Axis bivariate line-fitting analysis. The slopes are significantly different from each other (*p*-value = 0.00017), reflecting an evolutionary change affecting developmental trajectories of imaginal discs among different mountains since their isolation. **b** Winged queen larvae shows extensive cell division in imaginal discs in last larval instar. Wingless queen larvae from the Northern Sky Islands **c** Pinals and the **d** Catalinas show very few cell divisions in their vestigial wing discs while the Southern Sky Islands, the **e** Huachucas and **f** Chiricahuas show more. Red is PH3, green is DAPI, and yellow arrowheads indicate a cell that is expressing PH3. **g** Map of the Sky Islands indicating the position of Northern and Southern Sky Islands. **h** Graph of the number of dividing cells per unit area in vestigial discs of wingless queen larva from different Sky Islands. The x-axis shows the different mountain ranges and the y-axis the number of cells dividing per (*μ*m^2^). The number of dividing cells per unit area in vestigial wing discs differs between Northern and Southern Sky Islands (ANOVA, *p* <0.0001). Thus, the vestigial wing discs of queens from Southern Sky Islands show significantly higher levels of cell division and are larger than vestigial wing discs of queens from Northern Sky Islands

Based on this shared ancestral developmental potential for producing wingless queens in the genus *Monomorium*, as well as the very recent history shared by *M. emersoni* populations, we initially predicted that similar changes in the wing developmental system would underlie the parallel evolution of the wingless phenotype. Wings in holometabolous insects develop from imaginal discs that are clusters of cells in larvae [17]. These cells also give rise to the flight muscles and to the wing hinge during metamorphosis [20, 59–62]. In wingless queens, wing imaginal discs appear as vestigial remnants during the last larval instar (Additional file 1: Figure S5). We characterized the cell division and growth of wing imaginal discs and found that vestigial wing discs of wingless queen larvae from different mountain ranges significantly differ in size (Fig. 4) and levels of cell division (Fig. 4b to f). Because larvae were reared under the same experimental conditions, these differences are a consequence of an evolutionary change in growth, not developmental plasticity, in wingless queens across Sky Islands. Surprisingly, we discovered that these differences may be the consequence of a demographic split that happened ∼80,000 years BP between the northern (Catalinas and the Pinals) and the southern (Huachucas and the Chiricahuas) Sky Islands (Figs. 1e, 3a and 4g). Wingless queens from northern Sky Islands possess smaller discs and low levels of cell division (Fig. 4h), whereas those from the southern Sky Islands possess large imaginal discs and high levels of cell division. This association suggests that demographic events drove the evolution of novel variations in wing disc growth across *M. emersoni* populations.

**Fig. 5:**
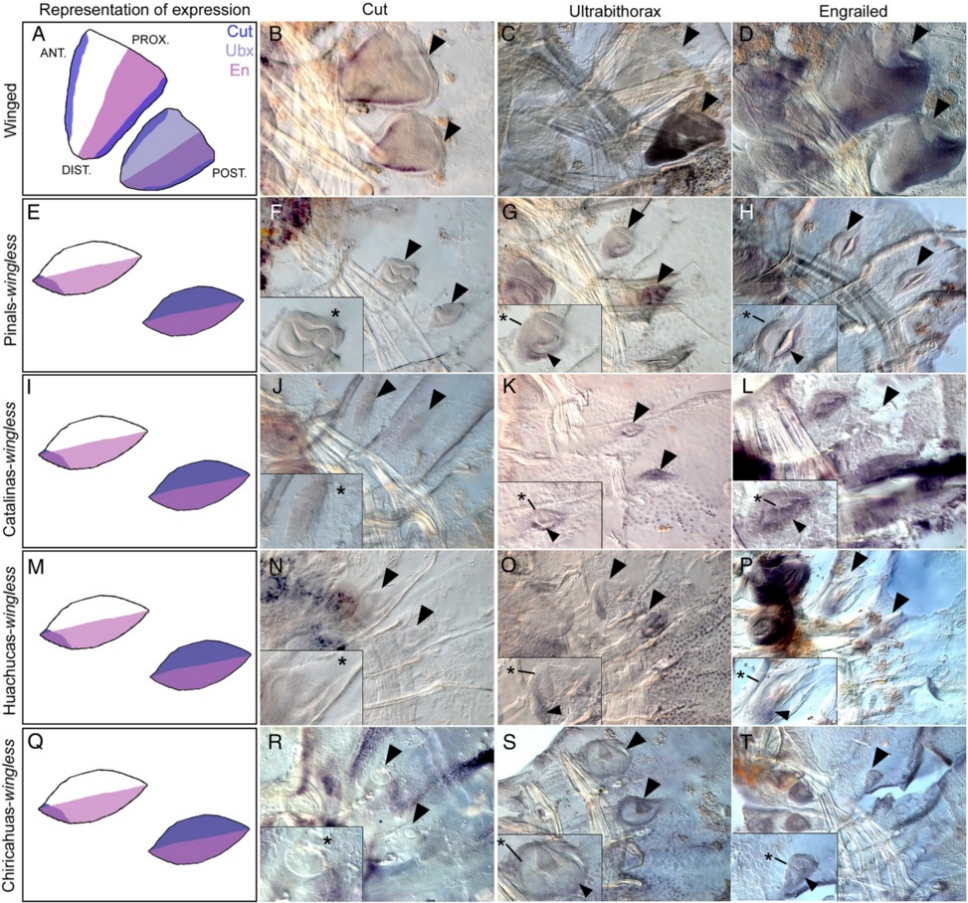
Expression profiles of *cut*, *Ubx*, and *en* in vestigial discs of wingless queens from different Sky Islands show similar expression patterns. Panels in the left column **a**, **e**, **i**, **m**, and **q** represent diagrams of expression patterns of Cut (blue), Ubx (violet) and En (pink). From left to right, columns show gene expression profiles (purple) of Cut (**b**, **f**, **j**, **n**, **r**), Ubx **c**, **g**, **k**, **o**, **s**, and En (**d**, **h**, **l**, **p**, **t**) in winged queens and wingless queens across four Sky Islands. Black arrowheads point to vestigial discs and asterisks indicate an absence of expression in the vestigial wing discs. Insets show forewing discs at higher magnification

**Fig. 6:**
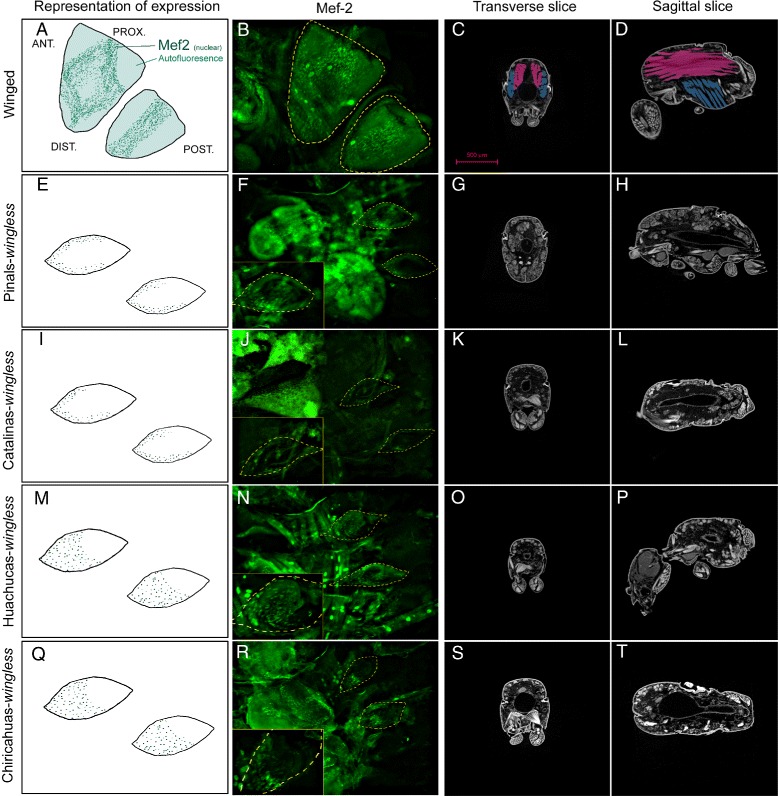
Wingless queen larvae develop wing muscle precursors, but lack flight muscles entirely. Panels on the left column (**a**, **e**, **i**, **m**, **q**) represent diagrams of Mef-2 expression. Mef-2 expression in larval myoblasts is shown as a localized stain in nuclei of myoblasts associated with the imaginal discs **b**, **f**, **j**, **n**, and **r**. Details on Mef-2 expression are given in Additional file 1: Figure S7. Inserts show forewing discs at higher magnification. Dashed lines on Mef2 expression patterns highlight the vestigial disc boundaries based on DAPI staining. Virtual sections (microCT) through the thorax (last two columns) showing the internal anatomy of a winged queen (top row) and wingless queens from all Sky Islands. **c**, **g**, **k**, **o**, and **s** show transverse sections and **d**, **h**, **l**, **p**, and **t** show sagittal sections. Longitudinal muscles are artificially colored in pink and dorso-ventral muscles in blue. Wingless queens from all Sky Islands show a complete absence of indirect flight muscles. These results show that, regardless of their origin, wingless queens do not develop the main flight muscles

**Fig. 7:**
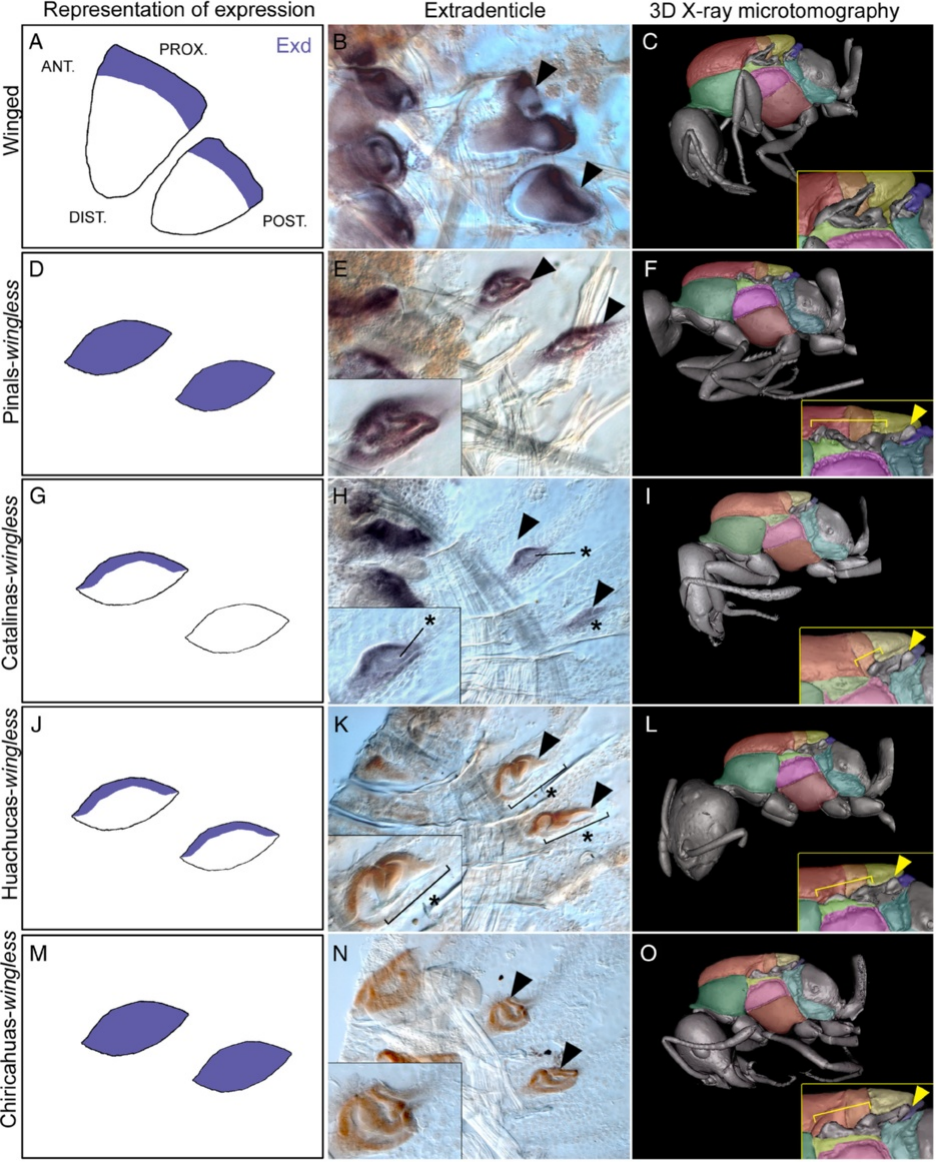
The variation in Exd expression pattern across Sky Islands corresponds to changes of fine details in thorax morphology of wingless queens. Panels on the left column (**a**, **d**, **g**, **j**, **m**) represent diagrams of Exd expression. Exd expression in winged queens **b** and wingless queens **e**, **h**, **k**, and **n**. Black arrowheads point to Exd expression in imaginal discs. Insets show a close-up of Exd expression in the vestigial imaginal discs. 3D X-ray microtomography thoracic morphology of a winged queen **c** and wingless queens from Pinals **f**, Catalinas **i**, Huachucas **l**, and Chiricahuas **o** Sky Islands. Insets show a close-up of the wing hinge region. Yellow brackets indicate vestigial forewing hinge and yellow arrowheads indicate outgrowths of the hindwing hinge. Note that the vestigial forewing hinge in **f** and **o** is prominent with two distinct outgrowths, while it is reduced in **i**, **l** with only one prominent outgrowth. The vestigial hindwing hinge is present in all populations

Wing discs express a gene network that controls growth and patterning of wings (Fig. 1f), of wing muscles, and of the wing hinge (Fig. 7, last column), and has been highly conserved across holometabolous insects for approximately 325 million years [20, 63–66]. In ants, the expression of this network is conserved in winged queens and males but is interrupted at specific points in the network in wingless workers [63]. These points of interruption in wingless workers are spatiotemporally associated with patterns of apoptosis in the vestigial wing disc [65]. We therefore determined whether points of interruption in this network in wingless queens has evolved in parallel or whether it differs between Sky Island populations. To confirm that expression of genes in this network is conserved in *M. emersoni* winged queens, we selected 6 genes within this network (Fig. 1f), including 4 genes involved in patterning wing tissue (*engrailed* [*en*], *Ultrabithorax* [*ubx*], *cut* [*cut*], *wingless* [*wg*]), one gene involved in wing muscle precursor development (*myosin enhancer factor 2* [*mef2*]), and one gene involved in wing hinge development (*extradenticle* [*exd*]). In winged queens, the expression of these genes is similar to those in the wing discs of *Drosophila* [20, 59–61] and winged queens and males of other ant species [63, 67] (Fig. 5b, c, and d, Fig. 6b, Fig. 7b and Additional file 1: Figure S6B), indicating that this network is conserved.

We then determined whether the expression of these genes has been interrupted in wingless queens, and found that their expression is altered relative to winged queens (Figs. 5, 6 and 7 and Additional file 1: Figure S6). In wingless queens from different Sky Islands, we found that the 4 genes involved in wing tissue patterning (*ubx*, *en*, *cut*, *wg*) show similar aberrant patterns of expression (Fig. 5e to t and Additional file 1: Figure S6C to H). These shared alterations reveal that wingless queens, regardless of their geographic origin and extent of vestigial wing disc growth (Fig. 4a), interrupt wing patterning at least in part through similar changes in gene expression. Therefore, although organ growth retains the signature of historical events caused by past demographic changes, alteration of the underlying expression of wing patterning genes is recurrent among populations on each Sky Island.

In contrast to genes involved in wing patterning, we observed that the expression of *mef2* - a key transcription factor involved in wing muscle development [61] - differs among populations and shows a signature of the demographic split between the southern and the northern Sky Islands. Mef2, which is expressed in the nuclei of myoblasts and appears as a dotted pattern in wing discs, shows expression differences between wingless queens from northern and southern Sky Islands (Fig. 6f and j versus N and R, and Additional file 1: Figure S7). In the northern Sky Islands, Mef2 expression is restricted to the outer edge of the vestigial disc in wingless queens, whereas in the southern Sky Islands, it is expressed more extensively and covers the central region of vestigial discs. Thus, like patterns of wing disc growth (Fig. 4h), Mef2 expression patterns precisely recapitulate the history of contacts between Sky Islands. Furthermore, these differences in the pattern of Mef2 expression in vestigial wing discs are likely to have evolved neutrally because wingless queens lack flight muscles regardless of their origin (Fig. 6g, h, k, l, o, p, s and t). This suggests that another mechanism, such as the interruption of other genes in the network or apoptosis of myoblasts [65], prevents *mef2* expressing cells from further differentiating into flight muscles.

**Fig. 8:**
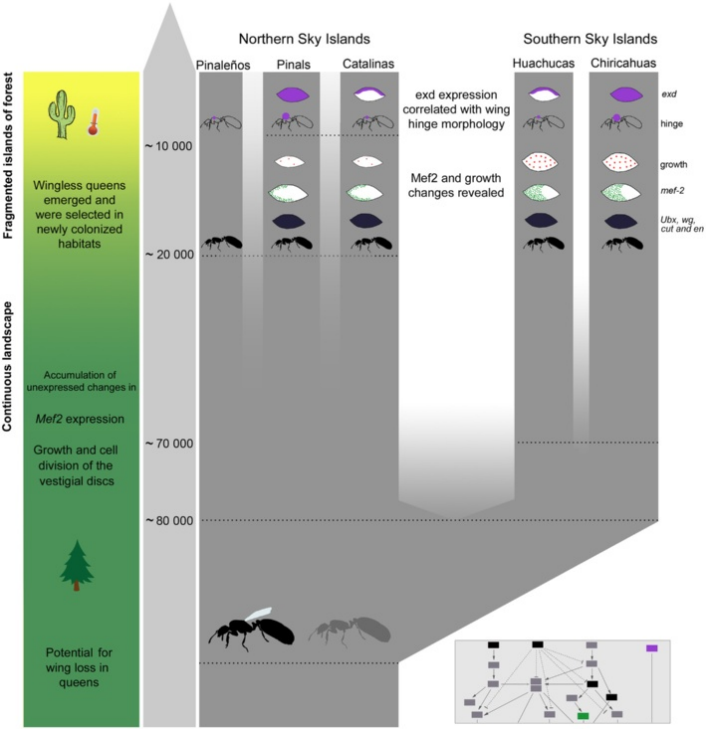
Summary of climatic changes, population history, wingless phenotype evolution and the associated changes in developmental systems in *M. emersoni*. The population tree represents the history of contacts during the last 200,000 years. Ancestral potential for wing loss (grey ant profile). Climatic changes are depicted in the bar on the left, with the associated changes in the habitats in different colors (green: largely continuous landscape, yellow: fragmented landscape). Changes in the vestigial disc growth, wing hinge morphology, wing muscles and in expression patterns of genes responsible for wing, wing hinge and wing muscle development are depicted by symbols on the tree. Gene expression on vestigial discs is depicted by purple (Exd), dotted green (Mef2) or black (wing patterning genes: Ubx, En, Cut and *wg*). Changes in cell division are depicted by the red dots

Finally, we found that *exd* - a gene known to be responsible for wing hinge development [20] - shows highly distinct expression patterns among wingless queens from different Sky Islands (Fig. 7b, e, h, k, and n) that correspond to fine differences in wing hinge morphology (Fig. 7c, f, i, l, and o). In wingless queens coming from the same Sky Island, the aberrant pattern of Exd expression is the same across individuals (Additional file 1: Figure S8). However, in wingless queens from the Pinals (north) and the Chiricahuas (south), Exd is expressed over the entire vestigial disc and adults have a prominent vestigial fore- and hindwing hinges, whereas in wingless queens from the Catalinas (north) and Huachucas (south), Exd is only expressed in the anterior edge of vestigial discs and adults have a prominent vestigial hindwing hinge but the forewing hinge is absent or dramatically reduced in size (Fig. 7 and Additional file 1: Table S6). These observed differences within northern (Pinals and Catalinas) and within southern (Chiricahuas and Huachucas) Sky Islands indicate that these differences arose following the complete isolation of the five Sky Island populations ∼10,000 years BP and are therefore an indirect consequence of climatic warming (Fig. 8).

## Discussion

### A paleoecological reconstruction for the parallel evolution of a developmental network and its phenotype in response to past climate change on Sky Islands

We have provided evidence for the following scenario, which shows that in response to both direct and indirect effects of past climate change on Sky Islands, a developmental gene network and its corresponding phenotype can respond to changing ecological conditions on short time scales (Fig. 8): Paleoecological reconstructions of the Arizona Sky Islands [24, 25] show that during the last glaciation between about 90,000 to 20,000 years BP, a continuous forest habitat across the landscape prevailed, except for high elevations which were inhospitable alpine habitats. The developmental potential to produce wingless queens in *M. emersoni* was present during this period. During or immediately following the vicariant event ∼80,000 years BP causing the demographic split between northern and southern Sky Islands, these *M. emersoni* populations accumulated unexpressed differences in the genes that control wing growth and flight muscle development. Subsequently, during climatic warming ∼10,000 years BP, the landscape became fragmented and new habitats arose at high elevations that were suitable for colonization by *M. emersoni*. As populations encountered these new habitats on each of the Sky Islands, the wingless phenotype evolved independently and became more frequent through selection on the phenotypic expression of the developmental potential for producing wingless queens. The parallel evolution of wingless queens on each Sky Island occurred through recurrent interruptions of similar wing-patterning genes in the network (*ubx*, *en*, *cut*, *wg*), and at the same time, induced the release of the unexpressed differences in vestigial wing growth and *mef-2* expression that accumulated in populations during or after the demographic split between the northern and southern Sky Islands ∼80,000 years BP. Climate warming during the last ∼10,000 years BP formed vast deserts between Sky Islands thereby isolating the five Sky Island populations from one another and forced *M. emersoni* populations to adapt physiologically to the warm conditions at the lower elevations of their range. This may have led to the rapid evolution of the vestigial wing hinge and its underlying *exd* expression on each Sky Island.

### Novelty as a mosaic of contingency and determinism

We show that developmental systems can facilitate two types of evolutionary change that contribute to the differentiation of populations within a species: one that is governed by chance events, is unpredictable, and has produced small differences in phenotype and genotype: wing hinge morphology, wing disc growth and expression of *exd* and *mef-2*. In contrast, the other type is repeatable and has produced large differences in phenotype and genotype: the wingless queen. The evolution of wingless queens represents an evolutionary novelty - it is the gain of a developmental switch, the complete loss of wings and wing muscles, multiple alterations in the expression of the core developmental network underlying wing development, and the gain of a new life history strategy (dependent colony foundation). These findings offer a new perspective for the ongoing discussion of novelty generation in phenotypic evolution [68]. On the one hand, it has been argued that adaptive solutions through natural selection are deterministic and that evolution is largely predictable [69], while on the other, it has been argued that historical contingency, which is the unique chain of prior events that determine which of several evolution paths a lineage will follow, renders evolution unpredictable [70]. Several studies, from a phylogenetically diverse array of organisms, show that the parallel evolution of phenotypic traits often occurs through recurrent alterations in the same genes [12, 23, 71–74]. Yet, at the same time, these and other studies report slight differences across multiple levels of biological organization: phenotype, development, and genetics [75–77]. In vitro studies on microbial populations suggest that these similarities and differences underlying the parallel evolution of traits are the consequence of a complex interplay between deterministic processes and historical events [78–80]. We show, in natural populations within a species, that the emergence of novelty at multiple biological levels can be generated by the combination of ancestral developmental potentials facilitating parallel evolution in response to similar ecological conditions and chance events that create slight variations on these repeatable evolutionary novelties.

A mosaic of contingent and deterministic processes at multiple biological levels may be a general property of the evolution of novelty. Most, if not all, organisms experience similar population dynamics over evolutionary time and parallel evolution within and between species is a nearly universal pattern throughout the tree of life [69, 81]. Many independently evolved traits, such as, pigmentation spots on *Drosophila* wings [53, 82], pharyngeal jaws of cichlid fish [83], hind limbs of anoles lizards [84], coat color of wild mice [85], and coloration on butterfly wings [86], exhibit patterns consistent with our findings in that they show slight variations in phenotype and genotype. Our results suggest that these slight variations are generated by microevolutionary forces and historical contingency acting between populations, together causing the gradual accumulation and evolution of unexpressed changes in regulatory pathways. These unexpressed differences become expressed during the process of parallel evolution leading to novel variations on independently evolved traits. This process driving parallel phenotypic novelty within species may translate into parallel phenotypic novelty between species, and as in the case pharyngeal jaws of cichlid fish [83], slight variations accumulated in one of the lineages may potentially fuel phenotypic diversification.

### Implications for bridging Micro- and Macroevolution

One of the biggest challenges facing evolutionary theory today is to explain how microevolution translates into macroevolution. Evolutionary biologists have addressed this problem from the perspective of paleontology [6, 87], population and quantitative genetics [88], and evolutionary developmental biology [16, 89, 90], producing a range of different perspectives on the translation between micro- and macroevolution. One perspective, which took hold during the neo-Darwinian synthesis in the 1940’s, proposes no distinction micro- and macroevolution, where macroevolution is simply the product of a specific kind of microevolution (mutations of small effect, individual level selection and drift) extrapolated over long temporal scales [88]. This perspective, however, is not well supported in the face of recent advances in quantitative genetics [8, 91], experimental evolution [92], developmental plasticity [3] and paleontology [93]. These recent advances show that: (1) dynamics of evolutionary change observed between individuals within populations cannot be extrapolated to explain the dynamics of change observed between species within clades [87, 93]; and (2) alleles of large effect [8, 91] and large discrete phenotypic variants [3, 54, 94] can be fixed in populations. Indeed, most modern textbooks of evolution now distinguish micro- from macroevolution and use the species boundary as the dividing line between them, most often defining microevolution as ‘evolution below the species level’ - small changes, adaptive or neutral, that arise within populations, while macroevolution is most often defined as ‘evolution above the species level’ - large changes that arise within populations leading to the origin of species or higher taxonomic groups [2, 95–98]. Although all changes must necessarily arise within populations, macroevolutionary changes are thought to arise *during* or *after* populations have undergone speciation, and therefore, characterize differences observed between closely-related species or higher taxonomic groups [3, 93].

Our findings, however, suggest that the species boundary may not adequately separate micro- and macroevolutionary changes. We observed macroevolution-like change (emergence of wingless queens) between populations within the same species (*M. emersoni*), which is comparable to the variation observed between species in the genus *Monomorium* (Table 2). Our observations are consistent with an idea known as ‘intraspecific macroevolution’, which proposes that micro- and macroevolution are distinct, but that speciation is not the primary cause of large phenotypic changes between species leading to macroevolution [3]. It proposes that both small and large phenotypic changes occur within the same species, and that the existence of alternative phenotypes within lineages facilitates macroevolution by reducing the negative fitness consequences associated with the appearance and fixation of large phenotypic variants [3].

Finally, it has been proposed that changes in hierarchically organized developmental networks can simultaneously produce the large- and small-scale changes associated with macro- and microevolution [99, 100]. Genes in the network involved in core developmental processes (‘kernels’ sensu Davidson, [18]), such as body plan formation, are thought to produce large-scale variants when changed, be highly stable during evolution, and are associated with macroevolution. In contrast, genes in the network involved in terminal differentiation processes, like pigment formation, are expected to produce small-scale variants, be more labile during evolution, and are associated with microevolution [99, 100]. Our results are consistent but show that the core developmental network responsible for wing formation facilitates the evolution of both types of changes within a species in response to ecological change. Based on all of our findings, we propose that the parallel phenotypic novelty within species may translate into parallel phenotypic novelty between species providing a possible bridge between micro- and macroevolution.

### The role of organismal development in past and future climate change

Understanding how developmental systems respond to past climate change is critical to facing the challenges of global warming over the next few decades. Integrating the responses of developmental systems with environmental changes will be necessary for building accurate projection models to predict species distributions under climatic change scenarios. Predicting species distributions accurately can affect decision making about conservation biology strategies, agriculture and public health issues. Current projection models integrate demographic processes such as migration and population size changes, but few integrate the possible response of developmental systems to environmental changes, which as we show, may profoundly affect the life history and dispersion abilities of a species. Ultimately, such changes directly or indirectly affect species interactions and ecosystem processes [101]. Sky Island mountain forests have been used as models and indicators of climate change. They have responded to past [24] and present [36] global warming by contracting towards the higher elevations [35], and climatic and ecological models predict that warming and desertification in Arizona will continue over the next 50 years [33, 34, 102]. Several species found in the mountainous areas of the American Southwest already show signs of changes in phenology in response to recent global warming [103, 104]. These dramatic changes in environmental conditions will be integrated by the developmental systems of different organisms, and as we show, can generate both predictable and unpredictable phenotypic responses. Generally integrating the predictable part of the response of developmental systems into climate change studies should hold the promise of making more accurate predictions for organismal responses to climate change.

## Conclusion

Since the modern synthesis, our view of how population differentiation occurs in the wild is largely based on the advances made in population and quantitative genetics, especially with the advent and integration of genomics tools. This view is one of changes in allele and phenotypic frequencies in response to adaptive and non-adaptive forces. By integrating organismal development and paleoecology on Sky Islands, we enrich this view by showing that the emergence of novelty within populations occurs through a combination contingent and deterministic processes. This combination results in the parallel evolution of traits with slight variations at multiple levels of biological organization (phenotype, growth, developmental networks). We propose that this parallel phenotypic novelty within the species may represent the springboards for translation into macroevolutionary changes between species. Studying these evolutionary processes on the Sky Islands, with their rich fauna provides replicated ecological gradients, are a good model system for understanding how development influences an organism’s response to past and future climate change.

## Materials and methods

### Animal collection and culturing

We discovered the *M. emersoni* wingless phenotype in the Chiricahuas mountain range in 1999. We found that this wingless phenotype exists on four other Sky Island mountains in Southeastern Arizona, USA and we collected colonies from 14 sites (Additional file 1: Table S3). We preserved colonies in 95 % ethanol or kept them alive in plastic boxes with glass test tubes filled with water constrained by a cotton plug. We fed colonies with a mix of crickets, mealworms and Bhatkar-Whitcomb diet [105]. The colonies were maintained at 27 ° C and 70 % humidity with a day/night cycle of 12 hours. We induced production of winged and wingless queens by exposing them to a cycle of temperature range: the temperature of the growth chamber was reduced by 1 ° C for 17 days until is reached 10 ° C. We kept the chamber at 10 ° C for 2 weeks, and then increased the temperature gradually by 1 ° C per day for 17 days until the original conditions were restored.

### Statistical analyses

We performed all statistical analyses using R, unless otherwise stated.

#### Inferring modern and historical population demography

##### Sequencing and genotyping

We extracted DNA from 318 *M.emersoni* queens, each from a different colony, using thoracic tissue from preserved individuals in ethanol. We amplified by PCR two mitochondrial genes (cytochrome oxidase 1 (CO1) and 2(CO2)), and sequenced them in both directions on an ABI3730 (for primers, see [106]) for a total of 1350 base pairs (bp) for 70 individuals. We also amplified and sequenced 1020 bp from a nuclear gene, *hymenoptaecin*, including one intron. For each Sky Island, we isolated alleles from *hymenoptaecin* to create a pool of DNA for each Sky Island by mixing DNA from about 20 queens from different colonies. This method has been shown to be accurate to estimate allele frequencies of a population [107, 108]. We amplified all alleles in the DNA pool simultaneously by PCR and cloned them into a PGEM-T vector. We picked 15 bacterial clones per mountain and we sequenced them with M13 primers in both directions. This allowed us to obtain the exact haplotype phase for every nuclear allele sampled and sequenced. All sequences were assembled and aligned with Geneious Pro V.5.4.3 and checked by eye. GenBank accession numbers are KT356282-KT356545.

We also genotyped all individuals for Amplified Fragment Length Polymorphisms (AFLP) markers, as described in [109]. We used 4 primer combinations to generate 157 unambiguous loci: *M*_*CTA*_ and *E*_*ACC*_, *M*_*CTA*_ and *E*_*ACG*_, *M*_*CAG*_ and *E*_*ACC*_, and *M*_*CAG*_ and *E*_*ACG*_. We ran the PCR products on a LiCor DNA analyser 4300 and the same researcher scored them blindly using the accompanying Saga software. To estimate the error rate in the procedure, about 10 % of the samples were amplified and scored twice, and we recovered a similar error rate to what has been reported in other studies [110].

##### Estimation of modern gene flow

We estimated *F*_*ST*_ values with the Bayesian method developed by [111] and implemented in AFLP-surv [112]. We removed from the analysis sites where we collected less than 15 individual queens from different colonies. *P*-values for the significance of each *F*_*ST*_ were estimated after applying Boneferroni corrections for multiple comparisons. Pairwise *F*_*ST*_ estimates for all 14 sites are reported in Additional file 1: Table S5. Stars represent parameters that could not be estimated with confidence. To test for the presence of recent gene flow, we performed a Mantel test [113] to calculate the correlation between the three-dimensional geographic distance and the genetic differentiation (*F*_*ST*_ estimates) for all pairwise comparisons among sites. We expected a significant correlation to occur if restricted but non-zero gene flow existed among Sky Islands, or if Sky Islands diverged recently from a continuous ancestral population without recent gene flow. On the other hand, we expected a non-significant correlation for the two following demographic scenarios: a complete panmixia among most sites, or a highly reduced gene flow for a long time among most sites. We tested the Mantel coefficient for significance using 10,000 random permutations on the matrices.

##### Estimation of historical gene flow and times of divergence

We used the Bayesian search strategy implemented in MIGRATE-n 3.2.15 [114] to estimate the effective population sizes, the average historical migration rates, the historical migration rates and the coalescence times for each population. We also evaluated 2 models of population subdivision by comparing their marginal likelihoods using the modified thermodynamic integration (TH) implemented in MIGRATE-n [115]. The first model considered all sampling sites as part of one panmictic unit while the second model considered each mountain range as independent units (limited or absence of gene flow). Each MIGRATE-n run consisted of 10 replicates of 4 MCMC heated short chains (heating scheme: 1.0, 1.2, 3.0, 6.0) and one long chain of 5,000,000 sampled trees where 500,000 burn-in trees were discarded to ensure proper sampling of the parameter space. All parameter estimates showed narrow posterior probability distributions, indicating high confidence in the estimates. All comparisons involving the Chiricahuas as the sending population show clear outlier values and were thus not considered. *Θ* values from the haploid and maternally-transmitted mitochondrial loci were corrected for comparison with a diploid locus to obtain an estimate of 4Ne *μ*. We used the direct estimate of mtDNA mutation rate calculated by [116] and the immune gene mutation rate estimate in *Drosophila* from [117] to calculate migration rates and times of divergence. MIGRATE-n also implements the Skyline plot method for detecting changes in migration rates over time, thus allowing the reconstruction of historical migrant exchanges between mountain ranges. The analysis was run separately for each pair of geographically adjacent mountain ranges (Chiricahuas-Huachucas, Huachucas-Catalinas, Catalinas-Pinals, Pinals-Pinaleños and Pinaleños-Chiricahuas) with the same run parameters as described above.

##### Testing for competing evolutionary scenarios of population splits

We used Approximate Bayesian Computation implemented in the program DIYABC v.1.0.4.45 beta [118] to test competing hypotheses of lineage divergence. Instead of estimating the likelihood of a model from an MCMC approach, DIYABC uses approximate Bayesian computation [119], where similarity of summary statistics between observed and simulated data sets are compared. Data can be simulated for several scenarios, where population sizes, times of divergence, population size change and admixture can be specifically defined.

In order to focus on testing the most probable scenarios, we built up our hypotheses based on the results of the Skyline plot analysis from MIGRATE-n (Additional file 1: Table S4 and Additional file 1: Figure S1). We compared three divergence scenarios; (A) a simultaneous isolation of the five mountain ranges from a common ancestor, (B) a sequential divergence of Sky Islands based on the results of the Skyline plot analysis using as priors the estimated times where no more gene flow is observed between two Sky Islands, and (C) a third scenario based on scenario B, but with the Huachucas and the Catalinas interchanged on the topology, thereby affecting the relationships within the Southern and Northern Sky Islands (Additional file 1: Figure S2).

The prior probability uniform distribution on the oldest divergence event (t4) ranged from 10,000 to 200,000 generations in the past and the prior probability uniform distribution on the effective population (Ne) size ranged from 1000 to 200,000 (coherent with MIGRATE-n estimates of Ne). The sequence of subsequent splits was forced to be kept in this specific order when simulating the data and the time interval prior probability distribution for each of these respective splits were t4 =1 to 50,000, t3 =10,000 to 70,000, t2 =25,000 to 100,000 and t1 =10,000 to 200,000 generations in the past. The prior on Ne was kept constant through time and was sampled from a uniform distribution ranging from 1000–100,000. Most importantly, three conditions were imposed for the sampling of parameters for the population splitting to occur in the specified order: t1 ≥ t2, t2 ≥ t3 and t3 ≥ t4. 300,000 simulations were performed and the direct estimate method was used to estimate the posterior probabilities of each scenario.

#### Parallel evolution of *M.emersoni* populations

##### Inferring mitochondrial haplotype network

To get insight about whether the wingless phenotype evolved repeatedly in parallel or has a single origin, we first infered genealogical realtionships among queen haplotypes across the Sky Islands populations. A mitochondrial DNA network was constructed using the R package *ape*. Divergent haplotypes (12 and 20 steps away from the central haplotype were discovered in the Chiricahuas but left out of the analysis for simplicity of the representation of the network.

##### Neighbor-joining tree

A neighbor-joining tree was constructed using AFLP data with the phylogenetic package Phylip [120]. The tree was constructed using sequentially the executables *Genedist*, *Neighbor*, *Seqboot*, *Consense* and *Drawtree*. Bootstrap values over 70 are reported on the tree.

##### Redundancy analyses

We first calculated the pairwise mtDNA genetic distance between each pairs of queens and performed a multidimensional scaling to obtain a set of synthetic variables that best represented the pairwise distances between records. To quantify habitat type in a discrete manner, we performed a principal component analysis using all environmental variables on all collection sites, assigning a habitat type (1 to 5) to sites with high similarity. We built three explanatory matrices: collection site, phenotype (winged or wingless) and environment (habitat type). We partitioned the variation in genetic distance with respect to the three explanatory matrices using a redundancy analysis ordination.

#### Adaptation to climatic changes

##### Tests for temperature tolerance

Heat resistance of worker ants was measured using a knockdown assay. About 20 individuals workers were collected from inside the nest of each colony, placed into 8 mL glass vials, and incubated at 50 ° C for one hour into a hybridization oven. Resistance was scored for each ant as the time taken to be knock down and mortality after treatment was recorded for each sample. Cold resistance was evaluated as recovery from a chill coma. About 20 individuals workers were collected from inside the nest of each colony, placed into small petri dishes, and immersed for 16 hours into a box filled with ice and water. This treatment led to rapid immobilization of all individuals. The temperature was kept constant around 4 ° C. Petri dishes were then exposed to ambient temperature and recovery was scored for each worker as the time taken for muscular coordination to come back.

#### Environmental factors the winged and wingless phenotypes may be adapting to along the ecological gradients

##### Explanatory variables for queen phenotype distribution

We calculated the percentage of wingless queens present at each site. This value represents the proportion of wingless queens that were successful in founding or persisting in a colony, and thus, have survived the dispersal/founding selection period. For each site, we measured the latitude and elevation in the field, and we obtained climatic variables from WorldClim Atlas and the vegetational growth average and vegetational growth peak were obtained from the National Atlas (Additional file 1: Table S1). The Worldclim Atlas and the National Atlas data have a 1 km^2^ resolution, which is, from our observations, of the same magnitude as the area of our average sampling sites.

We performed an information-theoretic approach to evaluate the performance of 12 models explaining the frequency distribution of winged and wingless queens along the ecological gradients. This approach, widely used in ecology, does not reject or accept models, but rather ranks a priori models and provides posterior probabilities that allow for the interpretation of the performance of each model. Using the findings of previous studies in the literature, we constructed 10 biologically plausible models that could explain which ecological factor(s) have the most influence on distribution of wingless queens along the ecological gradients. We used the method and R code of [121] to fit and rank models: we fitted models using the R function *lm*, and we calculated the sample-size corrected Akaike Information Criterion (AICc) for each model. We controlled for population differentiation using the score of the first component from a principal component analysis on the mtDNA pairwise genetic distances among all individual queens. We ranked the models based on AICc and calculated their posterior and conditional probabilities for further interpretation.

#### Interruptions of the gene network controlling wing development

##### Sample collection

We collected queen larvae at late last larval instar, just prior to the prepupal stage, following the criteria of [122]. We fixed larvae for 2h in a 4 % formaldehyde solution in PEM buffer [123], and dissected them under a Zeiss Discovery V12 Stereomicroscope to expose their thoracic imaginal discs by removing obstructive tissues.

##### Wing imaginal disc morphology and cell division

We calculated surface area of the forewing imaginal disc and of a leg imaginal disc (in *μ*m^2^) using AxioVision software (Carl Zeiss Canada Ltd., Toronto, Ontario, Canada). For all samples that were undamaged we counted the number of Phosphohistone expressing cells in the forewing imaginal disc from a z-stack image. To test for a difference in static allometry of leg and wing disc among populations, we performed a Standardized Major Axis bivariate line-fitting analysis (SAM) and tested for slope differences [124] (Additional file 1: Figure S6A). We also performed an analysis of variance on the number of cells undergoing mitosis per wing disc unit area for each population (Fig. 4).

##### Expression patterns of genes of the wing patterningnetwork

*Immunohistochemistry*: we performed antibody stainings for Engrailed (En), Ultrabithorax (Ubx), Cut (Cut) and Extradenticle (Exd) proteins following the protocol of [123]. Antibody stainings for Myosin enhancing factor-2 (Mef2) and Phosphohistone-3 (PH3) were conducted similarly, except that the tissues were treated with PAT 1 % instead of PTW 1 % to increase the antibody penetration in the tissue. We systematically included winged queen larvae in each staining reaction containing wingless queen larvae as positive controls and we over developed the staining reaction for larvae of both winged and wingless queens to confirm that the absence of expression was real and not an artifact. We imaged the stained tissues with a Zeiss microscope using the AxioVision software. Expression of genes in the network that controls wing development is highly conserved across winged castes of ants [63, 65]. Nonetheless, we confirm that winged queens from several collection sites do not show any expression differences (data not shown).

*Whole mount in-situ hybridization*: We isolated a fragment of the gene *wingless* by PCR using the primers of [63] on cDNA synthesized from embryonic and larval RNA. We cloned and sequenced this fragment to confirm its identity. Genbank accession number is KT361189. We followed the whole mount in-situ protocol from [125].

##### 3D X-ray microtomography

In order to describe in more detail the differences in external and internal anatomy of the thorax, we made 3D X-ray microtomography images (microCT scans) of winged queens and 11 wingless queens of different geographic origins. Ants were stained with elemental iodine (1 % w/v in absolute ethanol) to enhance the X-ray contrast of the soft tissues [126]. The scans were performed on an Xradia MicroCT 3D imaging system with voxel sizes between 1.8 and 4.2 *μ*m. We examined sagittal, coronal, and transverse sections for the presence of direct and indirect flight muscles. We applied a surface rendering algorithm using OsiriX to visualize the 3D reconstruction of the external anatomy of the thorax.

## Data availability

The population sets of DNA sequences generated in this study are available on GenBank (accession numbers KT356282-KT356545). Other data used in this study are available upon request to the authors.

## Additional file

Additional file 1
**Supplementary figures and tables.** This files includes supplementary tables and figures to the main manuscript. (PDF 4085kb)
