# Supplementary material for: Past climate change on Sky Islands drives novelty in a core developmental gene network and its phenotype
Source: BMC Evol Biol. 2015 Sep 4;15:183. doi: 10.1186/s12862-015-0448-4 (PMC4560157; doi:10.1186/s12862-015-0448-4)
Supplement: Additional file 1 — Supplementary figures and tables. This files includes supplementary tables and figures to the main manuscript. (PDF 4085kb) [file 12862_2015_448_MOESM1_ESM.pdf]

Supplementary tables and figures

Past climate change on Sky Islands drives novelty in a core developmental gene network and its phenotype

Marie-Julie Favé[1], Robert A. Johnson[2], Stefan Cover[3] Stephan Handschuh[4] Brian D. Metscher[4] Gerd B. Müller[4] Shyamalika Gopalan[1], Ehab Abouheif[1*]

1 Department of Biology, McGill University, 1205 Dr. Penfield avenue, Montréal, Qc, H3A 1B1, Canada

2 School of Life Sciences, Arizona State University, Tempe, AZ 85287-4501, USA

3 Museum of Comparative Zoology, Harvard University, 26 Oxford Street Cambridge, MA 02138, USA

4 Department of Theoretical Biology, University of Vienna, Althanstrasse 14, 1090, Vienna, Austria

∗ E-mail: ehab.abouheif@mcgill.ca

Supplementary Table 1

Environmental variables evaluated for each sampling site. Sky Islands are steep ecological gradients highly sensitive to environmental change. To characterize these gradients and evaluate the factors associated with frequency of the alternative queen phenotypes, we evaluated 12 biological models (Table 1) including combinations of these 5 ecological variables which were obtained from the different sources detailed below. The values of the variables obtained from Worldclim and The National Atlas were measured on a $1km^2$ grid map, which is just slightly larger than the average area for each sampling site. Therefore, the environmental variables are a reasonable proxy for the difference in the environmental conditions experienced by colonies at each site.

**Variable**	**Source**
Elevation	Measured in the field
Annual mean temperature	Worldclim
Temperature seasonality	Worldclim
Fragmentation class	The National Atlas
Productivity (Vegetation growth average)	The National Atlas

Supplementary Table 2

Elevation and fragmentation best explain the variation in frequency of wingless queens on the Arizona Sky Islands. Models are ordered by increasing values of the Akaike Information Criterion for small sample sizes (AICc). The number of parameters (k), the residual sum-of-square (RSS), the adjusted R^2, the corrected AIC (AICc), the model likelihood and the model probability are reported. The models are ordered from top to bottom from the highest to the lowest model probabilities (blue). Wingless queen frequency is best explained by a combination of habitat fragmentation and elevation. Model m06, which included the combined effects of fragmentation and elevation, shows by far the greatest model probability (0.58) and accounted for 50% of the variability in the data. The second best model includes habitat productivity as the explanatory variable and explains 22% of the variability, indicating that productivity of the habitat also has some predictive power for wingless queen distribution. Four of the five highest ranking models included fragmentation and/or elevation as explanatory variables, bringing additional support for an effect of these environmental variables on the frequency of wingless queens along the gradients. Subsequent models have very little empirical support and thus are not discussed further.

Rank	Model	Formula	k	RSS	Adjusted R^2	AICc	Model likelihood	Model Probability
1	m06	Fragmentation + Elevation	5	0.10	0.50	-12.34	1.00	0.56
2	m05	Productivity	4	0.17	0.22	-9.99	0.31	0.17
3	m04	Fragmentation	4	0.18	0.18	-9.16	0.20	0.11
4	m07	Fragmentation + Elevation + interaction	6	0.09	0.47	-6.55	0.06	0.03
5	m08	Temperature + Fragmentation	5	0.15	0.24	-6.50	0.05	0.03
6	m01	Elevation	4	0.21	0.00	-6.46	0.05	0.03
7	m03	Seasonality	4	0.22	-0.04	-5.83	0.04	0.02
8	m02	Temperature	4	0.23	-0.08	-5.40	0.03	0.02
9	m10	Fragmentation + Productivity	5	0.16	0.16	-5.15	0.03	0.02
10	m011	Fragmentation + Productivity + interaction	6	0.15	0.16	-0.18	0.00	0.00
11	m09	Temperature + Fragmentation + interaction	6	0.15	0.15	-0.03	0.00	0.00
12	m10	Structure rich	8	0.08	0.40	12.49	0.00	0.00

Supplementary Table 3

Sampling sites in the Arizona Sky Islands. We sampled fourteen sampling sites distributed over the ecological gradients of five Sky Islands for *M. emersoni* colonies. Sites are distributed along the ecological gradient of each Sky Island, from mid-elevations (around 1700m) to high elevations (over 2200m). From our observations, this range encompasses most areas inhabited by *M. emersoni*.

Site	Mountain range	Latitude	Longitude	Elevation (m)
Pinal-1	Pinals	33.30N	110.87W	1805
Pinal-2	Pinals	33.28N	110.82W	2345
Cat-1	Catalinas	32.45N	110.73W	2120
Cat-2	Catalinas	32.43N	110.75W	2443
Cat-3	Catalinas	32.43N	110.78W	2775
Hua-1	Huachucas	31.42N	110.26W	1700
Hua-2	Huachucas	31.43N	110.29W	2090
Chiri-1	Chiricahuas	31.88N	109.22W	1765
Chiri-2	Chiricahuas	31.77N	109.32W	1850
Chiri-3	Chiricahuas	31.93N	109.30W	1880
Chiri-4	Chiricahuas	31.93N	109.27W	2080
Chiri-5	Chiricahuas	31.90N	109.27W	2571
Pinos-1	Pinaleños	32.65N	109.81W	2025
Pinos-2	Pinaleños	32.70N	109.97W	2701

Supplementary Table 4

The number of migrants per generation between mountain ranges is low for both mtDNA and nuclear loci. Pairwise estimation of the number of migrants (Nm) per generation between mountain ranges for the *hymenoptaecin* locus and both mtDNA loci. The 2.5 and 97.5 confidence intervals on Nm are shown. For both nuclear and mtDNA loci, in all cases, the estimations of average Nm includes zero or values lower than one migrant per generation. Such values are generally considered as not hampering population differentiation. The marginal likelihood estimation supports a model of five isolated Sky Island populations rather than a panmictic population (likelihood of 1.0 for 5-populations-model versus 8.80×10^{-25} for panmictic population model). Therefore, we conclude that effective migrations between mountain ranges have been sufficiently restricted in the recent past to isolate all five Sky Islands. Our skyline plots show that connections or exchanges between populations were higher in the past (fig. S1)

				Nm per generation for nuclear locus- receiving mountain														
	Θ			Pinals			Catalinas			Huachucas			Chiricahuas			Pinaleños		
Mountain	2.5	mode	97.5	2.5	mode	97.5	2.5	mode	97.5	2.5	mode	97.5	2.5	mode	97.5	2.5	mode	97.5
Pinals	0.027	0.045	0.086	-	-	-	0	1.37	9.16	0	0.95	8.76	0	1.77	10.08	0	1.04	25.25
Catalinas	0.023	0.043	0.084	0	1.11	6.98	-	-	-	0	1.05	8.04	0	1.46	10.72	0	0.01	18.48
Huachucas	0.280	0.047	0.092	0	1.24	8.42	0	1.27	9.21	-	-	-	0	1.02	10.08	0	1.34	23.49
Chiricahuas	0.02	0.042	0.083	0	0.81	6.99	0	1.09	7.49	0	0.81	7.32	-	-	-	0	1.12	14.43
Pinaleños	0.009	0.026	0.069	0	0.01	5.04	0	0.37	4.31	0	0.30	5.18	0	0.01	4.76	-	-	-
				Nm per generation for mtDNA locus - receiving mountain														
	Θ			Pinals			Catalinas			Huachucas			Chiricahuas			Pinaleños		
Mountain	2.5	mode	97.5	2.5	mode	97.5	2.5	mode	97.5	2.5	mode	97.5	2.5	mode	97.5	2.5	mode	97.5
Pinals	0.001	0.013	0.040	-	-	-	0.01	0.86	9.18	0	0	6.97	0	0.29	2.55	0	0	7.30
Catalinas	0.001	0.017	0.044	0.01	0.91	9.96	-	-	-	0	0.30	8.71	0	0.43	3.10	0	0.32	8.71
Huachucas	0.005	0.021	0.057	0	0.89	11.51	0	0.86	9.18	-	-	-	0.04	0.68	4.98	0	0.52	11.37
Chiricahuas	0.031	0.049	0.074	*	*	*	*	*	*	*	*	*	-	-	-	*	*	*
Pinaleños	0.002	0.016	0.050	0	0.37	9.83	0.02	0.93	11.21	0	0.35	9.47	0	0.29	2.55	-	-	-

Supplementary Table 5

Genetic differentiation is high between Sky Islands but low within each Sky Island. Pairwise F_{ST} estimates between collecting sites as estimated with AFLP-surv. **Bold values** indicate a significant value after correcting for multiple comparisons (Bonferroni) and shaded values represent within-mountain comparisons. In general, F_{ST} estimates between two sites within the same Sky Island are lower (average = 0.06) than F_{ST} estimates between two sites from different Sky Islands (average = 0.13). Also, most F_{ST} estimates within a Sky Island are not statistically significant (except three comparisons within the Chiricahuas), while most F_{ST} estimates involving sites from different Sky Islands are statistically significant. This reveals a lack of genetic differentiation within each Sky Island and a moderate to strong genetic differentiation among Sky Islands. In addition, a Mantel test of the association between geographic distance and genetic differentiation was not significant (1000 permutations, p=0.86), indicating that drift, rather than gene flow, appears to be the dominant force in structuring genetic variation. Together, these results suggest that modern gene flow across Sky Islands is very low or absent, but that some level of gene flow exists between sites within each Sky Island, preventing any significant genetic differentiation to accumulate.

	Cat-1	Cat-2	Cat-3	Chi-1	Chi-2	Chi-3	Chi-4	Hua-1	Hua-2	Pinos-1	Pinos-2
Cat-1	-										
Cat-2	0.02	-									
Cat-3	0.05	0.06	-								
Chi-1	**0.14**	**0.09**	**0.07**	-							
Chi-2	**0.10**	**0.07**	**0.04**	0.04	-						
Chi-3	**0.10**	**0.08**	0.04	0.06	**0.12**	-					
Chi-4	0.04	**0.09**	**0.10**	**0.19**	**0.14**	0.03	-				
Hua-1	**0.13**	**0.11**	**0.13**	**0.19**	**0.20**	**0.16**	**0.20**	-			
Hua-2	**0.12**	**0.10**	**0.11**	**0.16**	**0.17**	**0.15**	**0.17**	0.01	-		
Pinos-1	**0.11**	**0.14**	**0.19**	**0.32**	**0.26**	**0.13**	**0.26**	**0.19**	**0.20**	-	
Pinos-2	**0.10**	**0.14**	**0.19**	**0.33**	**0.27**	**0.12**	**0.27**	**0.19**	**0.19**	0.03	-

Abbreviations: Cat: Catalinas, Chi: Chiricahuas, Hua: Huachucas, Pinos: Pinaleños. Numbers refer to sampling sites within each mountain range.

Supplementary Table 6

Frequency of wing hinge phenotypes differ in each Sky Island. Number of queens with each wing hinge phenotype in all mountain ranges. The reduced phenotype shows a wing hinge region with reduced structures and usually only two more pronounced outgrowths (Fig. 7 **I**), and sometimes with the presence of a reduced third outgrowth (Fig. 7 **L**). The prominent phenotype has more extensive three-dimensional structures and shows three well pronounced outgrowths on the thorax (Fig. 7 **F** and **O**). A Chi-square test shows that the frequency of each wing hinge phenotype is different between Sky Islands (all p-values < 0.05).

Mountain	Position	Reduced phenotype	Prominent phenotype
Pinals	North	2	15
Catalinas	North	18	2
Pinalenos	North	20	4
Huachucas	South	8	1
Chiricahuas	South	1	17

Supplementary Figure 1

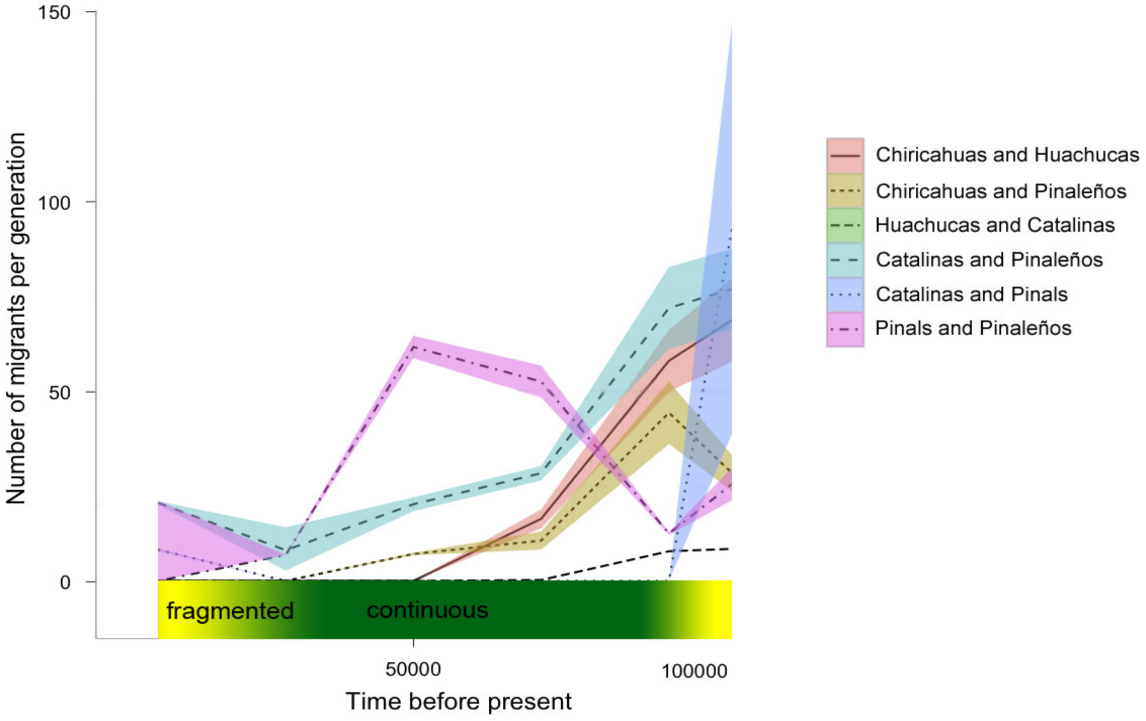

Graph comparing the number of migrants per generation (Nm) between pairs of adjacent Sky Islands (i.e.: Chiricahuas and Huachucas; Chiricahuas and Pinalenos; Huachucas and Catalinas; Catalinas and Pinals; Catalinas and Pinalenos; Pinals and Pinalenos). Estimates are averages of Nm from both directions, and colored shades represent 2.5 and 97.5 confidence intervals on the estimation of Nm. The lower bar represent the type of landscape (fragmented or continuous) and habitat (forest or desert dominated) inferred from climatic reconstructions of the Pleistocene habitats (reviewed in the introduction of the main text). We used these estimates as priors in a bayesian analysis to propose different evolutionary scenarios and test their probability (fig. S2).

Supplementary Figure 2

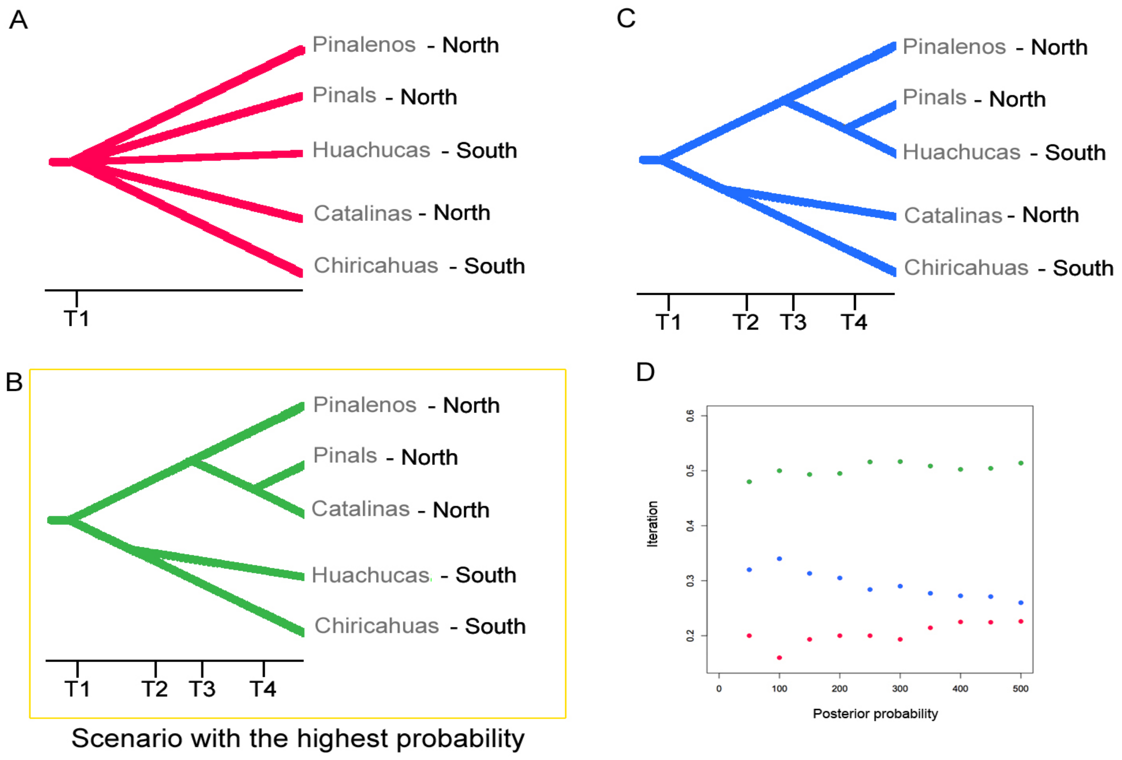

Evolutionary scenarios tested with an Approximate Bayesian Computation method confirms a scenario of sequential isolation of the five Sky Islands. Three evolutionary scenarios tested with DIYABC (see methods): (**A**) simultaneous isolation scenario of the five Sky Islands, (**B**) sequential splitting scenario of populations with the MIGRATE-n gene flow estimates used as priors (fig. S1) and (**C**) sequential splitting scenario of populations with Huachucas and Catalinas interchanged compared to scenario B. (**D**) shows the posterior probabilities for each scenario. Priors on time of divergence are described in the methods. These results support scenario B, where five Sky Islands have been sequentially isolated from each other over the last 200,000 years, with a first split occurring between the Northern and Southern Sky Islands, and then further division of the Sky Islands coinciding with climatic cycles during the Pleistocene.

Supplementary Figure 3

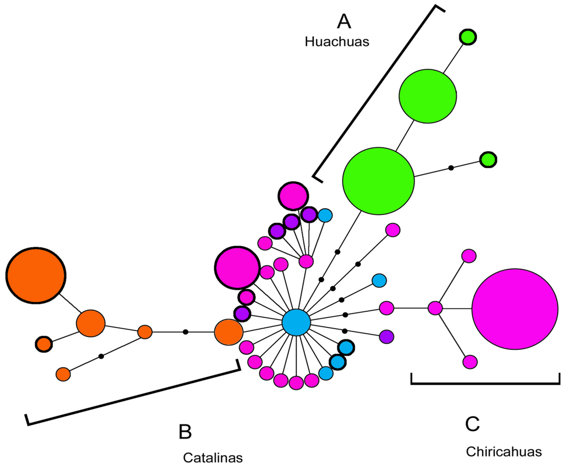

A mtDNA haplotype network reveals monophyly of populations on Sky Islands and is consistent with the parallel evolution of the wingless phenotype. mtDNA haplotype network, where colors identify Sky Islands: Pinals (blue), Catalinas (orange), Huachucas (green), Chiricahuas (pink), and Pinaleños (purple). The size of the haplotype representation is proportional to the number of individuals carrying this haplotype. Black dots represent mutational steps for which no haplotype has been detected, bold circles represent the wingless queens individuals. These results show that genetic structure reflects mostly geography as no haplotypes were shared between any two Sky Islands. Some Sky Islands have achieved monophyly (Huachucas (**A**) and Catalinas (**B**)) and some groups of related haplotypes are specific to some sites on a Sky Island (Chi-1 (**C**)). We find that populations on each Sky Island contain unique haplotypes variants, while no shared haplotypes were recovered among Sky Islands, indicating they originated within each Sky Island. These results show, in combination with the RDA analysis (Fig. 3B), that geography predominates in the patterns of genetic differentiation at neutral loci. This suggests an incipient process of parallel diversification of the populations. This scenario of incipient diversification is further supported by the tree recovered from AFLP analyses (fig. S4).

Supplementary Figure 4

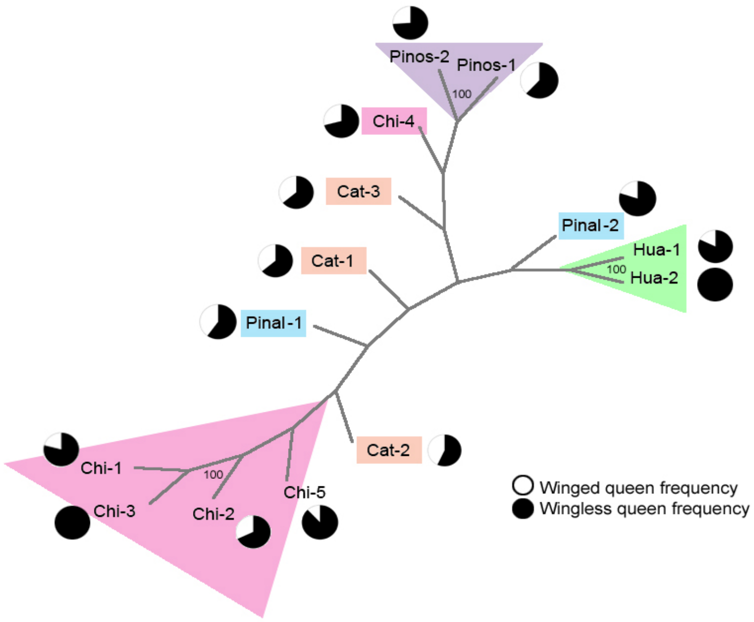

Phylogenetic analyses on AFLPs show that *M. emersoni* colonized each Sky Island independently A single origin of the wingless phenotype would indicate that wingless queens did not evolve independently on each mountain and predicts that queens would group according to the phenotype. Multiple origins of the wingless phenotype would indicate parallel evolution of the wingless phenotype on each mountain and predicts that queens would group according to the geography. The colors identify Sky Islands as follow: Pinals (blue), Catalinas (orange), Huachucas (green), Chiricahuas (pink), and Pinaleños (purple). Pie charts represent the proportion of winged (white) and wingless (black) queens at each site. Neihgbor-joining tree based on Nei's genetic distance from AFLP data showing that the sites from the Huachucas, the Chiricahuas, and the Pinaleños form groups that are highly supported (except for one site in the Chiricahuas). These groupings show that most of the genetic variation is explained by geography, and not habitat or phenotype. These analyses supports the hypothesis of multiple origins of the wingless phenotype, where genetic variation is structured by geography rather than habitat, phenotype (see also Fig. 3B) or elevation.

Supplementary Figure 5

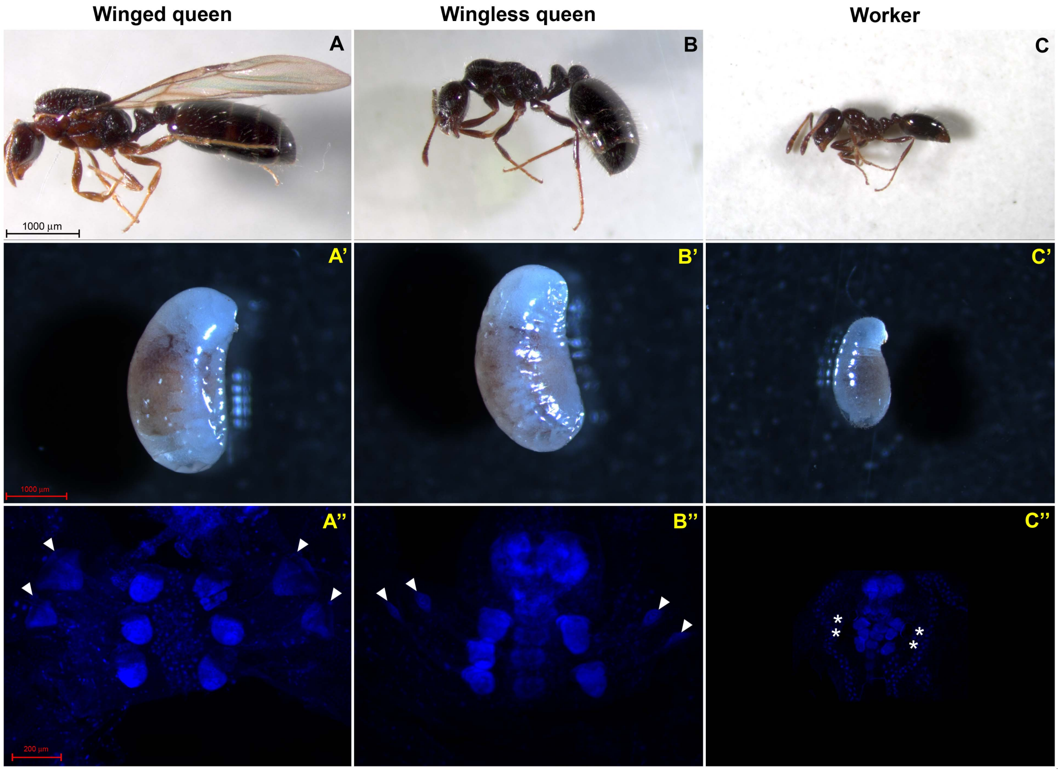

Development of *M.emersoni* winged queen, wingless queen, and worker. *M.emersoni* adult specimens of a winged queen (**A**), a wingless queen (**B**) and a worker (**C**). Last larval instar of a winged queen (**A'**), wingless queen (**B'**) and worker (**C'**). At the last larval instar, larvae from queen and worker are easy to discriminate from each other (compare **A'** and **B'** to **C'**). By dissecting third instar larvae and staining them for DAPI (blue), we can distinguish larvae that will develop into a winged queen (**A"**), a wingless queen (**B"**) or a worker (**C"**). White arrowheads indicate wing imaginal discs of the winged queen (**A"**) and vestigial wing imaginal discs in the wingless queen (**B"**). Asterisks indicate the expected positions of vestigial fore- and hindwing primordia in workers (**C"**). Adult, larval, and vestigial wing disc images are to scale and the same magnification is used for each of the horizontal groups of panels.

Supplementary Figure 6

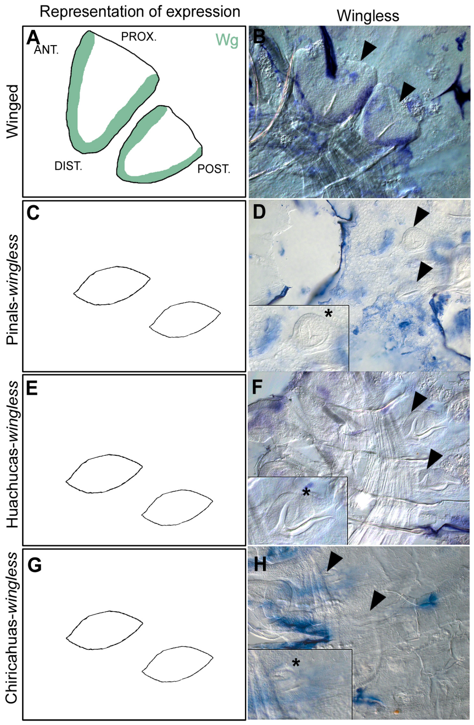

Expression of *wg* gene across Sky Islands Winged queen larva expression patterns **B** are conserved relative to those of winged castes of other ants and *Drosophila*. In wingless queens, wg is not expressed, regardless of the origin of the queens (**D**, **F**, and **H**). *wg* expression in vestigial discs from wingless queen larvae in the Catalinas is missing because we exhausted all samples from this location. Asterisks indicate an absence of expression and small black arrowhead indicate expression. All images are to scale.

Supplementary Figure 7

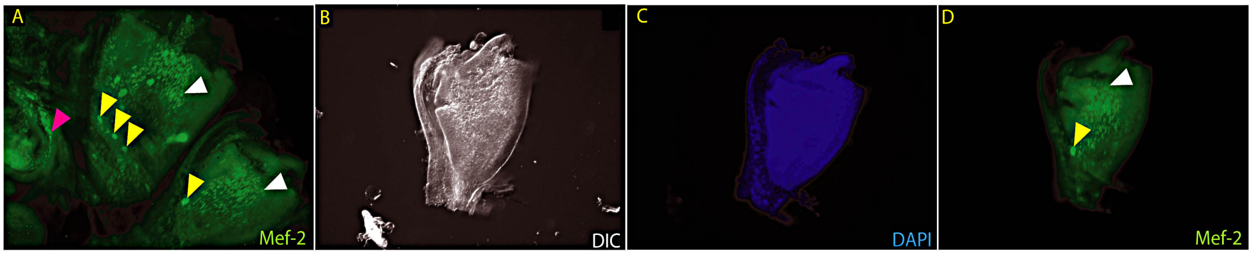

***mef2* expression in myoblasts associated with imaginal discs is distinguishable from expression in larval sarcomeres.** (**A**) Wing and leg imaginal discs expressing *mef2* (green). To distinguish Mef2 between imaginal disc myoblasts and larval muscles sarcomeres, we examine the Mef2 expression in a dissected larvae (**A**) and the same wing disc detached from the larval cuticle (**B** to **D**). Wing imaginal disc in DIC (**B**) and DAPI (**C**) and GFP fluorescence (**D**). Expression in myoblasts associated with imaginal discs is observed in nuclei. White arrowheads indicate to some myoblasts as examples on wing imaginal discs, while pink arrowhead indicates myoblasts on leg imaginal discs, and yellow arrowheads point to *mef2* nuclear expression in larval muscle sarcomeres. These are usually large and arranged in rows, and the muscle fiber is often also observable. A larval sarcomere in a muscle fiber is still attached to the disc in (**D**) (yellow arrowhead). On panels (**A**) and (**D**), the uniform green signal (not localized in the cellular nuclei) is due to autofluorescence.

Supplementary Figure 8

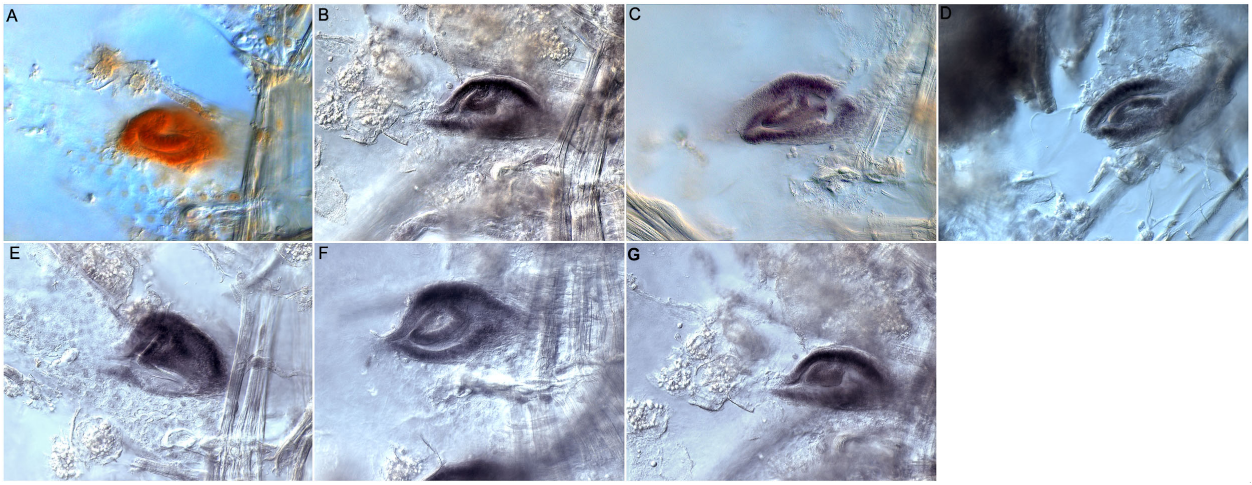

Exd expression is similar across individuals from the same Sky Island. We examined several samples within a Sky Island (Pinals) to test the assumption that variation within a Sky Island is lower than across Sky Islands. We chose *exd* because it shows the most variation across Sky Islands of all the other genes we sampled. Red or purple shows Exd expression. All images are to scale.
